# Vγ4 T Cells Inhibit the Pro-healing Functions of Dendritic Epidermal T Cells to Delay Skin Wound Closure Through IL-17A

**DOI:** 10.3389/fimmu.2018.00240

**Published:** 2018-02-12

**Authors:** Yashu Li, Yangping Wang, Lina Zhou, Meixi Liu, Guangping Liang, Rongshuai Yan, Yufeng Jiang, Jianlei Hao, Xiaorong Zhang, Xiaohong Hu, Yong Huang, Rupeng Wang, Zhinan Yin, Jun Wu, Gaoxing Luo, Weifeng He

**Affiliations:** ^1^State Key Laboratory of Trauma, Burn and Combined Injury, Institute of Burn Research, Southwest Hospital, Third Military Medical University (Army Medical University), Chongqing, China; ^2^Department of Endocrinology, Southwest Hospital, Third Military Medical University (Army Medical University), Chongqing, China; ^3^Chongqing Key Laboratory for Disease Proteomics, Department of Endocrinology of Southwest Hospital, Chongqing, China; ^4^Wound Healing Center, Medical School of Chinese PLA, Chinese PLA General Hospital, Beijing, China; ^5^Biomedical Translational Research Institute, Guangdong Province Key Laboratory of Molecular Immunology and Antibody Engineering, Jinan University, Guangzhou, China; ^6^Department of Dermatology, Xinqiao Hospital, Third Military Medical University (Army Medical University), Chongqing, China; ^7^Department of Burns, The First Affiliated Hospital, Sun Yat-Sen University, Guangzhou, China

**Keywords:** wound healing, Vγ4 T cells, dendritic epidermal T cells, IL-17A, IL-1β, IL-23, IGF-1

## Abstract

Dendritic epidermal T cells (DETCs) and dermal Vγ4 T cells engage in wound re-epithelialization and skin inflammation. However, it remains unknown whether a functional link between Vγ4 T cell pro-inflammation and DETC pro-healing exists to affect the outcome of skin wound closure. Here, we revealed that Vγ4 T cell-derived IL-17A inhibited IGF-1 production by DETCs to delay skin wound healing. Epidermal IL-1β and IL-23 were required for Vγ4 T cells to suppress IGF-1 production by DETCs after skin injury. Moreover, we clarified that IL-1β rather than IL-23 played a more important role in inhibiting IGF-1 production by DETCs in an NF-κB-dependent manner. Together, these findings suggested a mechanistic link between Vγ4 T cell-derived IL-17A, epidermal IL-1β/IL-23, DETC-derived IGF-1, and wound-healing responses in the skin.

## Introduction

γδ T cells are important components of the skin immune system and participate in psoriasis (1), graft rejection (2), carcinoma (3), antimicrobial barrier function (4), and wound repair (5). Several subsets of γδ T cells with distinctive functions exist in skin tissue: dendritic epidermal T cells (DETCs), which uniformly express an invariant Vγ5Vδ1 TCR (according to Heilig and Tonegawa’s nomenclature) and exclusively reside in the murine epidermis; Vγ1 and Vγ4 T cells, two dominant subsets of murine peripheral γδ T cells, have also been found to distribute in the dermal layer of murine skin (6). DETCs are rapidly activated at wound sites after injury and provide a major source of epidermal IGF-1, which is required for efficient wound repair (7). Vγ1 and Vγ4 T cells play distinctive roles in the allergic response (8), autoimmune diseases (9, 10), antitumor responses (11, 12), as well as infectious immunity (13, 14). However, whether and how Vγ1 and/or Vγ4 T cells affect wound healing remains unknown.

IL-17A is an important pro-inflammatory cytokine that plays a critical role in the initiation and amplification of inflammation responses. At the early stages of inflammation, IL-17A is primarily derived from γδ T cells, though it is secreted by Th17 cells at late stages of inflammation (1). IL-17A is required for efficient skin wound healing, as *Il-17a*^−/−^ mice exhibit defects in wound repair (4). In line with this notion, DETCs have been identified to provide a source of epidermal IL-17A after skin injury, which accelerates wound healing by inducing epidermal keratinocytes to express the host-defense molecules β-defensin 3 and RegIIIγ (4). Vγ4 T cells have been identified as a major source of IL-17A that plays a role in skin diseases (1, 15). Moreover, our previous studies have shown that IL-17A production was impaired in skin around wounds, which explains the impaired wound healing in diabetic settings, and IL-17A-positive Vγ4 T cells transferred to the wound bed could improve diabetic wound healing (16). However, Rodero MP et al. reported a contradictory role of IL-17A in skin wound repair and found that application of an IL-17A neutralizing antibody (Ab) onto the wound bed could significantly promote wound healing (17). Recently, using a skin transplantation model, we showed that Vγ4 T cells were recruited to the epidermis from the dermis in a CCL20-CCR6-dependent manner and primarily provided IL-17A at the wound edge at early stages of transplantation (18). The precise roles of IL-17A and Vγ4 T cells in skin wound healing still need to be clarified.

The IL-1β/IL-23-IL-17A axis is critical for the initiation and amplification of inflammatory responses (9, 19–22). IL-1β and IL-23 can strongly promote γδ T cells to produce IL-17A in γδ T cell-mediated skin diseases (2, 9, 21, 22). Our previous study revealed that IL-1β and IL-23 were required for Vγ4 T cells to accelerate skin graft rejection (18) and that DETCs could upregulate IL-17A production in response to IL-1β plus IL-23 upon TCR engagement (4, 22). IL-1β and IL-23 in the epidermis are mainly derived from keratinocytes and Langerhans cells (9, 22). Intriguingly, the expression of epidermal IL-1β and IL-23 could be enhanced by IL-17A derived from Vγ4 T cells in the transplantation area (18). However, whether and how the IL-1β/IL-23-IL-17A axis affects the engagement of γδ T cells in wound healing needs to be investigated.

IGF-1 is exclusively produced from DETCs in the epidermis and strongly promotes keratinocyte proliferation and migration for efficient wound healing (7). Although IL-17A secreted by DETCs has also been shown to improve skin wound closure (4, 7), IGF-1 plays a much more important role for DETCs in wound repair than IL-17A. The underlying molecular mechanisms and how the interaction of IL-17A and IGF-1 affects wound healing are important questions that need to be answered.

In this study, we observed that Vγ4 T cells were a major source of epidermal IL-17A at the early stages of wounding and were responsible for the delayed wound repair. Moreover, we highlighted that Vγ4 T cell-derived IL-17A indirectly inhibited IGF-1 production in DETCs by enhancing epidermal IL-23/IL-1β expression.

## Results

### Vγ4 T Cells Delay Skin Wound Healing *via* DETCs

**Figure 1:**
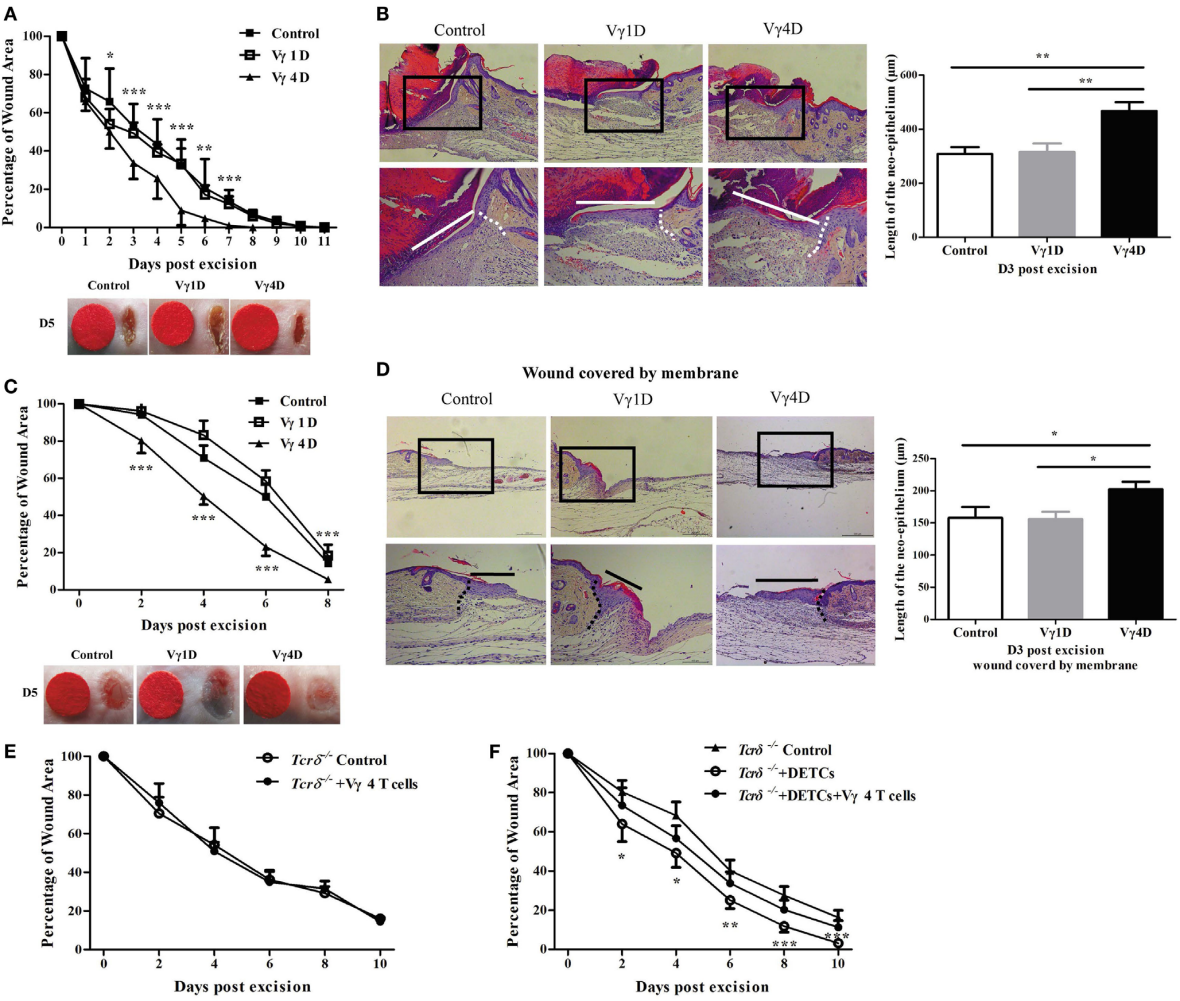
Vγ4 T cells have a negative impact on skin wound healing. **(A–D)** Age- and sex-matched C57BL/6 wild-type mice were intraperitoneally injected with 200 μg/mouse Vγ1 T-cell depletion (Vγ1D) Ab 2.11, Vγ4 T-cell depletion (Vγ4D) Ab UC3, or isotype control Ab (Control) on day 3 before wound excision. Full-thickness wounds were then generated using a sterile 6 mm punch tool on day 0. Wound-closure kinetics was measured over time in wound model with contraction **(A)** or without contraction **(B)**. Stars mark the comparations between Vγ4D and Control in **(A,B)**. On day 3 post-wounding, re-epithelialization in wound model with contraction **(C)** or without contraction **(D)** was analyzed by HE (*n* = 5–7). **(E)** Fresh isolated Vγ4 T cells (1 × 10^4^ cells/wound) or PBS were added onto wound bed of *Tcrδ*^−/−^ mice immediately after wound excision. **(F)** Fresh isolated dendritic epidermal T cells (DETCs) (5 × 10^4^ cells/wound), DETCs (5 × 10^4^ cells/wound) plus Vγ4 T cells (1 × 10^4^ cells/wound), or PBS was added onto wound bed of *Tcrδ*^−/−^ mice immediately after skin injury. Wound-closure kinetics was measured over time, and dots in wound closure kinetics represent 10 wounds per group (2 wounds/mouse, 5 mice/group). Stars mark the comparations between *Tcrδ*^−/−^ mice + DETCs + Vγ4 T cells and *Tcrδ*^−/−^ control. The values were calculated as the mean ± SD. All data represent at least three independent experiments. *P* value was calculated by Student’s unpaired *t*-test **(A,C,E,F)** or one-way ANOVA with Bonferroni’s multiple comparison test **(B,D)** (*p < 0.05, **p < 0.01, ***p < 0.001).

To investigate whether Vγ1 and Vγ4 γδ T cells are involved in skin wound repair, we used a murine wound model with or without contraction and analyzed the cutaneous wound-healing kinetics in age- and sex-matched C67BL/6 wild-type (WT) mice with Vγ1 or Vγ4 T cell depletion treatment [Vγ1 T-cell depletion (Vγ1D) or Vγ4 T-cell depletion (Vγ4D)]. The results showed that mice with Vγ4D compared to isotype controls displayed markedly improved wound healing (Vγ4D vs. control, wound model with contraction, Figure 1A; wound model without contraction and Figure 1C) and re-epithelialization (wound model with contraction, Figure 1B; wound model without contraction and Figure 1D), while mice with Vγ1D treatment showed similar results to controls (Figures 1A–D), indicating that Vγ4, but not Vγ1 T cells, could delay wound healing. However, the addition of freshly isolated Vγ4 T cells onto the wound bed of *Tcrδ*^−/−^ mice failed to affect skin wound repair of *Tcrδ*^−/−^ animals (Figure 1E), suggesting that Vγ4 T cells indirectly affected wound healing.

Because DETCs play a crucial role in skin wound closure (7), we assessed the contribution of DETCs in delayed wound healing mediated by Vγ4 T cells. The wounds of *Tcrδ*^−/−^ mice were supplemented with freshly isolated DETCs alone or DETCs with Vγ4 T cells. Supplementing DETCs onto the wound bed markedly improved wound repair in *Tcrδ*^−/−^ mice (*Tcrδ*^−/−^ + DETCs vs *Tcrδ*^−/−^ control, Figure 1F), but the improvement was notably attenuated by the addition of Vγ4 T cells (Figure 1F). This finding indicated that DETCs are essential for Vγ4 T cell-delayed skin wound closure.

### Vγ4 T Cells Inhibit IGF-1 Production in DETCs to Affect Skin Wound Healing

**Figure 2:**
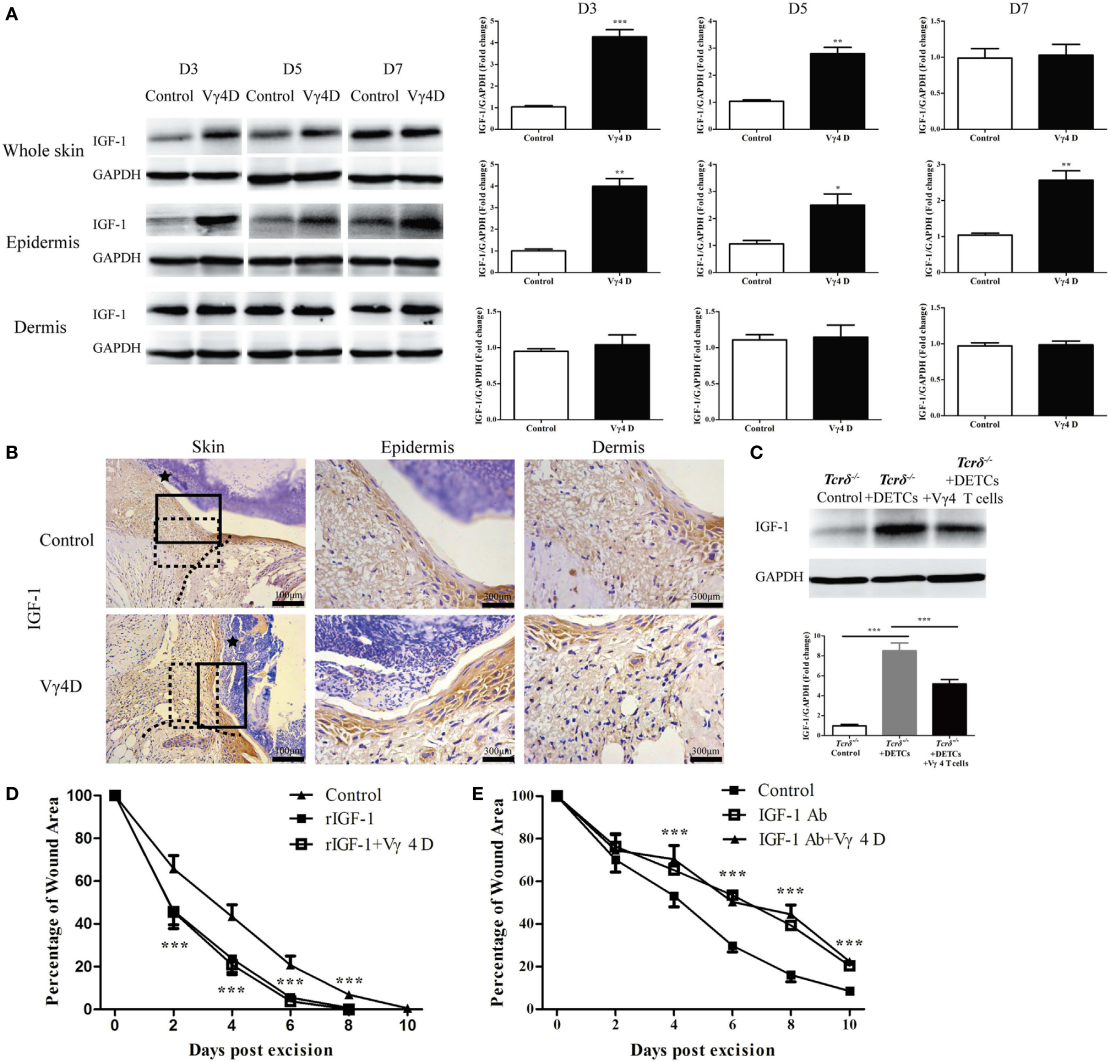
Vγ4 T cells inhibited epidermal IGF-1 production to delay skin wound repair. **(A,B)** Full-thickness wounds were generated in Vγ4 T-cell depletion (Vγ4D) and control mice. On day 3, 5, and 7 post-wounding, each group of epidermis tissues at wound margins was collected from at least three mice for further analysis of IGF-1 production by means of WB **(A)** and IHC **(B)**. The area of wound and neo-epidermis is marked by black star (Skin). Neo-epidermis in solid rectangles and neo-dermis in dashed rectangles were amplified threefolds and labeled as Epidermis and Dermis **(B)**. **(C)** Fresh isolated dendritic epidermal T cells (DETCs) (5 × 10^4^ cells/wound), DETCs (5 × 10^4^ cells/wound) plus Vγ4 T cells (1 × 10^4^ cells/wound), or PBS were added onto wound bed of *Tcrδ*^−/−^ mice (*n* = 5/group) immediately after skin injury, respectively. IGF-1 expression in epidermal wound margin was detected by WB on day 3 post-wounding. **(D,E)** Age- and sex-matched C57BL/6 wild-type mice were intraperitoneally injected with Vγ4D Ab UC3, or isotype control Ab (Control) on day −3. Followed by administration of 200 ng/wound rIGF-1 **(D)** or 20 μg/wound IGF-1 neutralizing Ab **(E)** onto wound margin on days 0, 1, and 2 post excision. Control mice in **(E)** were treated with IGF neutralizing isotype Ab (poly goat IgG). Wound-closure kinetics was measured over time and dots in wound closure kinetics represent 10 wounds (2 wounds/mouse, 5 mice/group). Star-like markers represent the differences between rIGF-1 + Vγ4D and Control in **(D)**, IGF-1 Ab + Vγ4D and Control in **(E)**. Data are representative of at least three independent experiments with cohorts of the indicated number of mice per group. Bars show mean ± SD. *P* value was calculated by Student’s unpaired *t-*test **(A,D,E)** or one-way ANOVA with Bonferroni’s multiple comparison test **(C)** (***P < 0.001).

IGF-1 is exclusively secreted by DETCs in the epidermis and is important for the pro-healing function of DETCs (7). Mice with Vγ4D exhibited markedly enhanced IGF-1 production in the epidermis but not the dermis around wounds compared to control animals (WB, Figure 2A; IHC, Figure 2B). Furthermore, transferring DETCs onto wound beds notably improved IGF-1 production in the epidermis around wounds of *Tcrδ*^−/−^ mice, but the enhancement was significantly attenuated when Vγ4 T cells were added (Figure 2C). Therefore, we considered that Vγ4 T cells inhibited IGF-1 expression in DETCs. Wound healing of WT mice was accelerated by rIGF-1, and wound healing of Vγ4D mice was also promoted when rIGF-1 (200 ng/wound) was supplemented onto wound beds (rIGF-1 + Vγ4D vs. control, Figure 2D). Anti-IGF-1 neutralizing Ab (20 μg/wound) markedly delayed wound healing not only in WT but also in Vγ4D mice (IGF-1 Ab + Vγ4D vs. control, Figure 2E). According to the results in Figures 2D,E, no differences between WT and Vγ4D mice were observed when intervening IGF-1 expression in the epidermis, suggesting that Vγ4 T cells may depend on IGF-1 to affect wound repair.

### Vγ4 T Cells Suppress IGF-1 Production but Not the Number and Activation of DETCs

**Figure 3:**
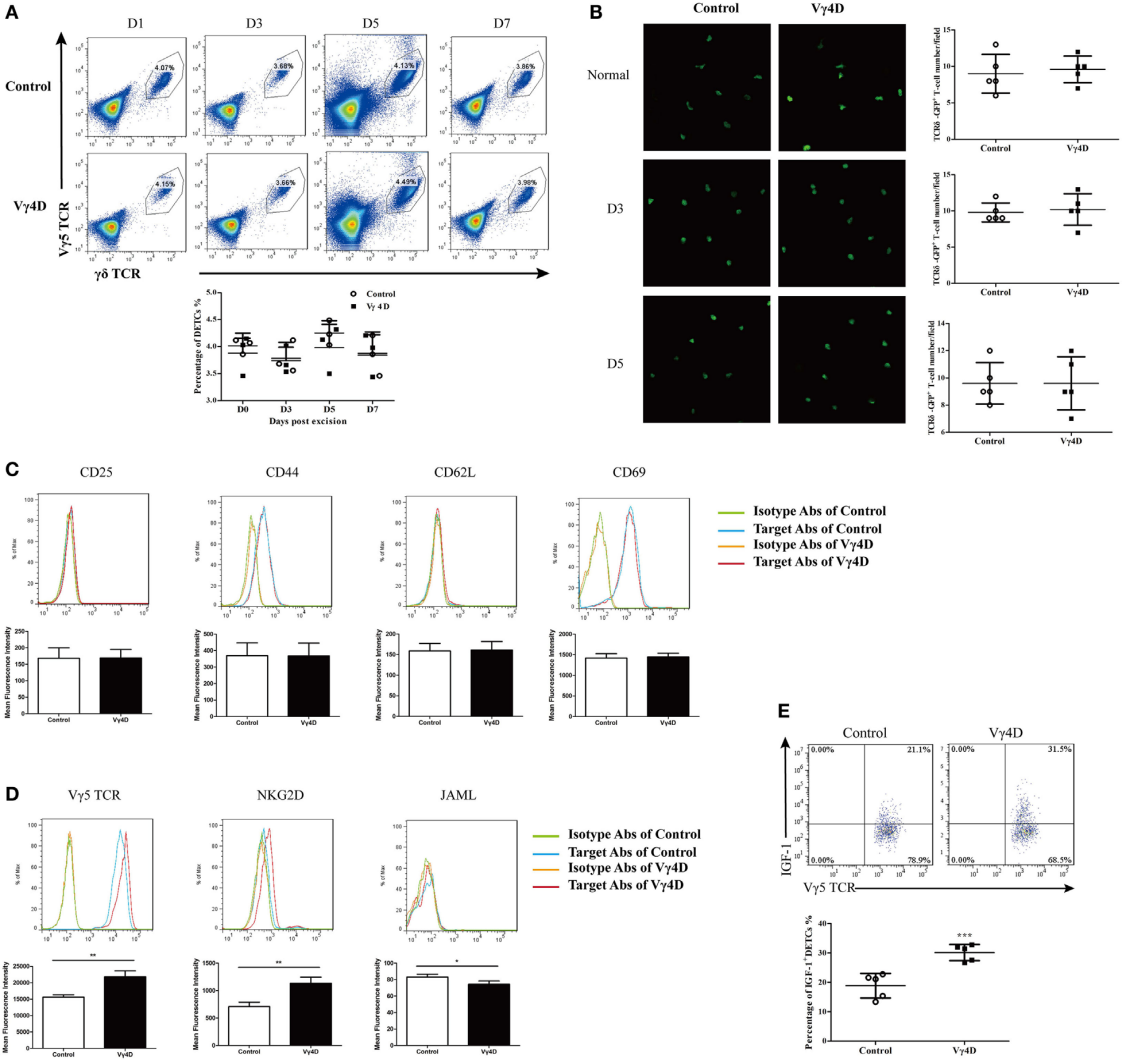
IGF-1 production by dendritic epidermal T cells (DETCs) around wound area was enhanced in Vγ4 T cell depletion mice. **(A)** The percentages of Vγ5 T cells in epidermis at wound margin of Vγ4 T-cell depletion (Vγ4D) and control mice were detected by FACS on days 0, 3, 5, and 7 after wounding. Statistical histogram is showed on the right panel. **(B)** Age- and sex-matched TCRδ-GFP mice were intraperitoneally injected with Vγ4D Ab UC3 or isotype control Ab (Control) on day −3. Epidermal sheets were separated from skin at wound margin of Vγ4D and control TCRδ-GFP mice on days 0, 3, and 5 after wounding. The morphology (left panel) and numbers/field of view (right panel) of DETCs around the wounds in Vγ4D and wild-type (WT) control mice were analyzed by confocal microscopy. **(C–E)** Epidermal cells were isolated from skin at wound margin of Vγ4D and WT control mice on day 3 post wounding. Expressions of T cell activation markers (CD25, CD44, CD62L, and CD69) **(C)** and T cell activation receptors (Vγ5 TCR, NKG2D, and JAML) **(D)** on surface of DETCs were analyzed by FACS. **(E)** The fresh isolated epidermal cells were stimulated with PMA (50 ng/ml) and ionomycin (750 ng/ml) for 6 h in the presence of Brefeldin A (100 ng/ml). IGF-1 productions by DETCs were then analyzed by FACS. Error bars represent mean ± SD. Each group of epidermis tissue around wound was gathered from 3 to 6 mice (4 wounds/mouse). All data were representative of three individual experiments. *P* value was calculated using One-way ANOVA with Bonferroni’s comparison test **(A)** or Student’s unpaired *t*-test in **(B,E)** (***P < 0.001).

Epidermal IGF-1 is primarily derived from DETCs (7), and Vγ4 T cells may decrease epidermal IGF-1 expression by decreasing number, impairing activation, and/or altering IGF-1 production by DETCs. We identified that Vγ4D could not affect the ratios (Figure 3A) and numbers (Figure 3B) of DETCs located around the wounds after skin injury. Furthermore, the expression of surface activation markers, such as CD25, CD44, CD62L, or CD69, on DETCs around wounds was comparable between Vγ4D and WT controls (Figure 3C). Interestingly, Vγ4D could influence the expression of activation receptors on DETCs. With the exception of JAML, TCR δ was slightly increased, and NKG2D was markedly enhanced on DETCs around the wounds (Figure 3D). However, IGF-1 production by DETCs at the wound margin was significantly increased in Vγ4D mice compared with WT controls upon PMA plus ionomycin stimulation *ex vivo* (Figure 3E). Therefore, the underlying mechanisms of Vγ4 T cells inhibiting epidermal IGF-1 production were likely down-regulating IGF-1 production rather than impacting the number or activation of DETCs *in vivo*.

### IL-17A Is Required for Vγ4 T Cells to Delay Wound Repair

**Figure 4:**
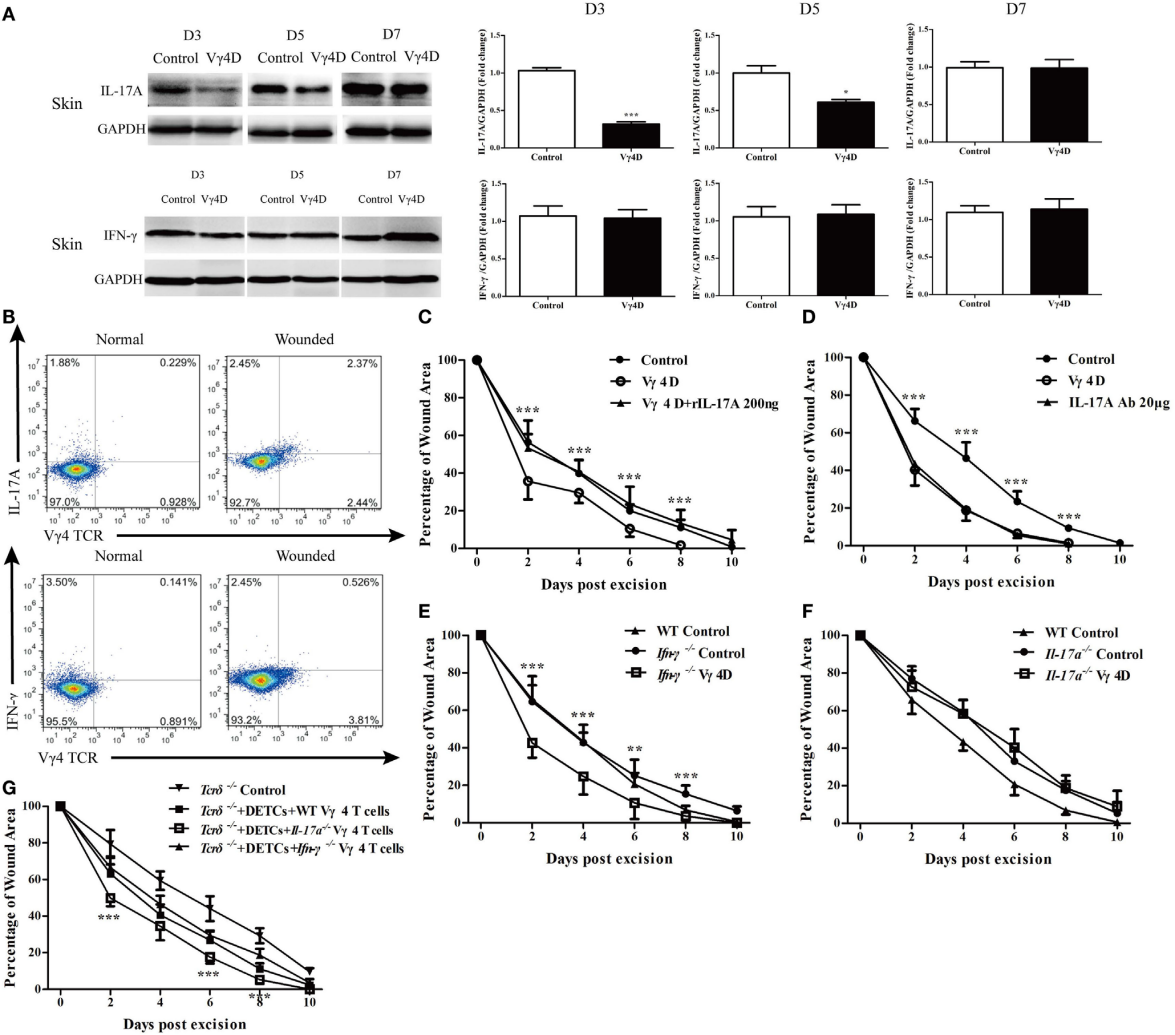
IL-17A was required for Vγ4 T cell-mediated delay of skin wound healing. **(A)** The expressions of IL-17A and IFN-γ in skin around wound of Vγ4 T-cell depletion (Vγ4D) and wild-type (WT) control mice were analyzed by WB on days 3, 5, and 7 after skin injury (*n* = 3–5/group). **(B)** The IL-17A and IFN-γ production by Vγ4 T cells around wound of Vγ4D and WT control mice were analyzed by FACS on day 3 post excision (4 wounds/mouse, 3–5 mice/group). **(C,D)** Age- and sex-matched C57BL/6 WT mice were intraperitoneally injected with Vγ4D Ab UC3, or isotype control Ab (Control) on day −3. Followed by administration of 200 ng/wound rIL-17A into wound margin of Vγ4D mice **(C)** or 50 μg/wound IL-17A neutralizing Ab into wound margin of WT mice **(D)** on days 0, 1, and 2 post excision. Control mice in **(D)** were treated with IL-17A-neutralizing isotype Ab (mouse IgG1). Wound-closure kinetics was measured over time and dots in wound closure kinetics represent 10 wounds (2 wounds/mouse, 5 mice/group). Stars represent the comparations between Vγ4D and Vγ4D + rIL-17A in **(C)**, IL-17A Ab and Control in **(D)**. **(E,F)** Age- and sex-matched *Ifn-γ*^−/−^ **(E)** and *Il-17a*^−/−^ **(F)** mice were intraperitoneally injected with Vγ4D Ab UC3, or isotype control Ab on day −3. Full-thickness wounds were then generated on day 0. Wound-closure kinetics was measured over time and dots in wound closure kinetics represent 10 wounds (2 wounds/mouse, 5 mice/group). WT mice were treated with isotype Ab as WT control. Stars represent the differences between *Ifn-γ*^−/−^ Vγ4D and *Ifn-γ*^−/−^ Control in **(E)**. **(G)** Fresh isolated dendritic epidermal T cells (DETCs) (5 × 10^4^ cells/wound) plus WT Vγ4 T cells (1 × 10^4^ cells/wound), DETCs (5 × 10^4^ cells/wound) plus *Il-17a*^−/−^ Vγ4 T cells (1 × 10^4^ cells/wound), DETCs (5 × 10^4^ cells/wound) plus *Ifn-γ*^−/−^ Vγ4 T cells (1 × 10^4^ cells/wound), or PBS was added onto wound bed of *Tcrδ*^−/−^ mice immediately after skin injury. Wound-closure kinetics was measured over time and dots in wound closure kinetics represent 10 wounds per group (2 wounds/mouse, 5 mice/group). Stars represent the comparations between *Tcrδ*^−/−^ + DETCs + *Il-17a*^−/−^ Vγ4 T cells and *Tcrδ*^−/−^ + DETCs + Vγ4 T cells in **(G)**. Bars represent mean ± SD. All data were representative of three independent experiments. *P* value was calculated by Student’s unpaired *t*-test (*P < 0.05, **P < 0.01, ***P < 0.001).

Vγ4 T cells provide a significant early source of IFN-γ and IL-17A to regulate immune responses (12, 15). It was observed that production of IL-17A was significantly reduced, whereas IFN-γ production was equally distributed in the skin around wounds of Vγ4D mice compared to controls (Figure 4A). Relative to the results, IL-17-positive Vγ4 T cells occupied nearly half of the total Vγ4 T cells in the epidermis around wounds, while IFN-γ-positive Vγ4 T cells were less than 5% (Figure 4B). Moreover, Vγ4D was shown to accelerate wound repair in Figure 1, but the application of rIL-17A (200 ng/wound) on wound beds with Vγ4D nearly eliminated the improvement of wound healing mediated by Vγ4D (Vγ4D + rIL-17A vs Vγ4D, Figure 4C). The administration of IL-17A neutralizing Ab (20 μg/wound) on wound beds significantly improved wound healing in WT mice (IL-17A Ab vs. control, Figure 4D), which was similar to the kinetic curve of Vγ4D animals (Figure 4D). In addition, Vγ4D in *Ifn-γ*^−/−^ mice markedly accelerated skin wound closure compared to *Ifn-γ*^−/−^ mice with isotype controls (*Ifn-γ*^−/−^ Vγ4D vs *Ifn-γ*^−/−^ control, Figure 4E). However, the wound-healing kinetics was analogous between *Il-17a*^−/−^ mice with Vγ4D and *Il-17a*^−/−^ mice with isotype control (Figure 4F). Compared to *Tcrδ*^−/−^ controls, the addition of WT DETCs together with *Il-17a*^−/−^ Vγ4 T cells onto wound beds facilitated wound healing compared to WT Vγ4 T cells (*Tcrδ*^−/−^ + DETCs + *Il-17a*^−/−^ Vγ4 T cells vs. *Tcrδ*^−/−^ + DETCs + WT Vγ4 T cells, Figure 4G), while *Ifn-γ*^−/−^ Vγ4 T cells did not contribute to wound repair compared to WT Vγ4 T cells (Figure 4G). These findings demonstrated that IL-17A, not IFN-γ, was responsible for Vγ4 T cell-delayed wound closure.

### Vγ4 T Cells Provide an Early and Significant Source of Epidermal IL-17A and Infiltrate into the Epidermis Depending on the CCR6-CCL20 Pathway after Skin Injury

**Figure 5:**
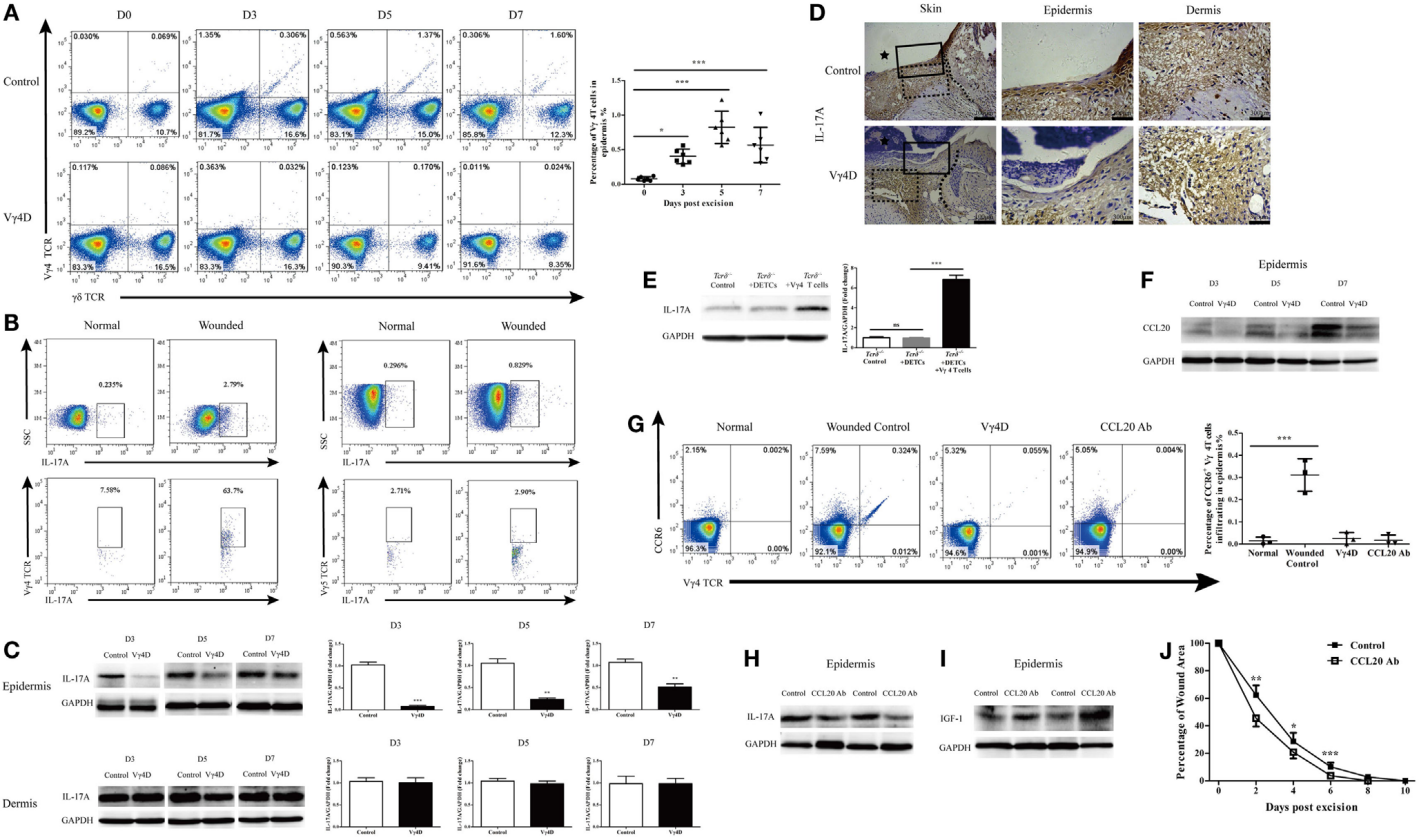
Vγ4 T cells *via* CCR6-CCL20 pathway infiltrated into epidermis and provided major early source of epidermal IL-17A after wounding. **(A)** Vγ4 T cells in epidermis around wound of Vγ4 T-cell depletion (Vγ4D) and wild-type (WT) mice were analyzed by FACS on days 0, 3, 5, and 7 after skin injury (4 wounds/mice, 5–7 mice/group). **(B)** Epidermis infiltrating IL-17A-positive cells around wound of WT mice (4 wounds/mice, 3–5 mice/group) were analyzed by FACS on days 0 and 3 after skin injury (upper panel). Gated on IL-17A-positive cells, the percentage of Vγ4 T cells and dendritic epidermal T cells (DETCs; anti-Vγ5 TCR) are shown (lower panel). **(C)** The expression of IL-17A in epidermis around wound of Vγ4D and WT mice was analyzed by WB on days 3, 5, and 7 after skin injury (*n* = 3–5/group). **(D)** The expression of IL-17A in epidermis around wound of Vγ4D and WT mice was analyzed by IHC on day 3 post-excision (*n* = 3–5/group). The area of wound and neo-epidermis is marked by black star (Skin, left panel). Neo-epidermis in solid rectangles and neo-dermis in dashed rectangles are shown on the middle (Epidermis) and right (Dermis) panel. **(E)** Fresh isolated WT DETCs (5 × 10^4^ cells/wound), Vγ4 T cells (1 × 10^4^ cells/wound), or PBS was added onto wound bed of *Tcrδ*^−/−^ mice immediately after skin injury (*n* = 3/group). On day 3 post-excision, the production of IL-17A in epidermis around wound was determined by WB. **(F)** The expressions of CCL20 in epidermis around wound of Vγ4D and WT control mice were detected by WB on days 3, 5, and 7 after wounding (*n* = 3/group). **(G)** Addition of CCL20 neutralizing Ab (10 μg/per wound) onto wound bed of Vγ4D and WT control animals at day 0, 1, and 2 after skin injury. On day 3 post-excision, the expressions of CCR6 on surface of Vγ4 T cells in epidermis around wound of animals with Vγ4D, CCL20 neutralizing, or control isotype Abs treatment were detected by means of FACS (4 wounds/group, 3 mice/group). **(H–J)** Addition of CCL20 neutralizing Ab (10 μg/wound) or isotype Ab (rat IgG1) into wound margin on days 0, 1, and 2 after skin injury. The production of IL-17A **(H)** and IGF-1 **(I)** in epidermis around wound was analyzed by WB on day 3 after wounding (*n* = 3–5/group). **(J)** Wound-closure kinetics was measured over time and dots in wound closure kinetics represent 10 wounds per group (2 wounds/mouse, 5 mice/group). Bars represent mean ± SD. All data were representative of three independent experiments. *P* value was determined by one-way ANOVA with Bonferroni’s comparision test **(A,E,G)** or Student’s unpaired *t*-test **(C,J)** (*P < 0.05, **P < 0.01, ***P < 0.001).

As previously mentioned, Vγ4 T cells suppressed wound healing by downregulating IGF-1 production in DETCs. In the skin, Vγ4 T cells and DETCs are physiologically distributed in the dermis and epidermis, respectively. It is reasonable to assume that Vγ4 T cells infiltrated into the epidermis after wounding and thus directly interacted with DETCs. Indeed, the percentage of Vγ4 T cells was significantly increased in the epidermis (Figure 5A), decreased in the dermis, and unchanged in draining lymph nodes after wounding (Figure S1 in Supplementary Material). Additionally, more than 50% of the infiltrating IL-17A-positive cells in the epidermis were Vγ4 T cells, and less than 5% of those were DETCs on day 3 after wounding (Figure 5B). Moreover, Vγ4D resulted in dramatically reduced IL-17A production in the epidermis rather than the dermis (WB, Figure 5C; IHC, Figure 5D). Supplementing with freshly isolated Vγ4 T cells rather than DETCs markedly enhanced epidermal IL-17A production around wounds in *Tcrδ*^−/−^ mice (Figure 5E). Therefore, we concluded that Vγ4 T cells infiltrated the epidermis around wounds to provide an early, major source of epidermal IL-17A after skin injury.

The CCR6-CCL20 pathway is critical for Th17 to infiltrate into the inflamed area (23). The production of epidermal CCL20 was markedly enhanced after skin injury (Figure 5F), and nearly all of the Vγ4 T cells that infiltrated the epidermis were CCR6-positive (Figure 5G). Moreover, blocking CCL20 with neutralizing Ab dramatically declined the ratio of infiltrating Vγ4 T cells (Figure 5G) and weakened epidermal IL-17A production (Figure 5H) but enhanced epidermal IGF-1 production (Figure 5I), followed by significantly accelerated skin wound repair (Figure 5J). These results demonstrated that Vγ4 cells that infiltrated into the epidermis rely on the CCL20-CCR6 pathway after skin injury.

### IL-17A Downregulated Epidermal IGF-1 Production at the Wound Margin

**Figure 6:**
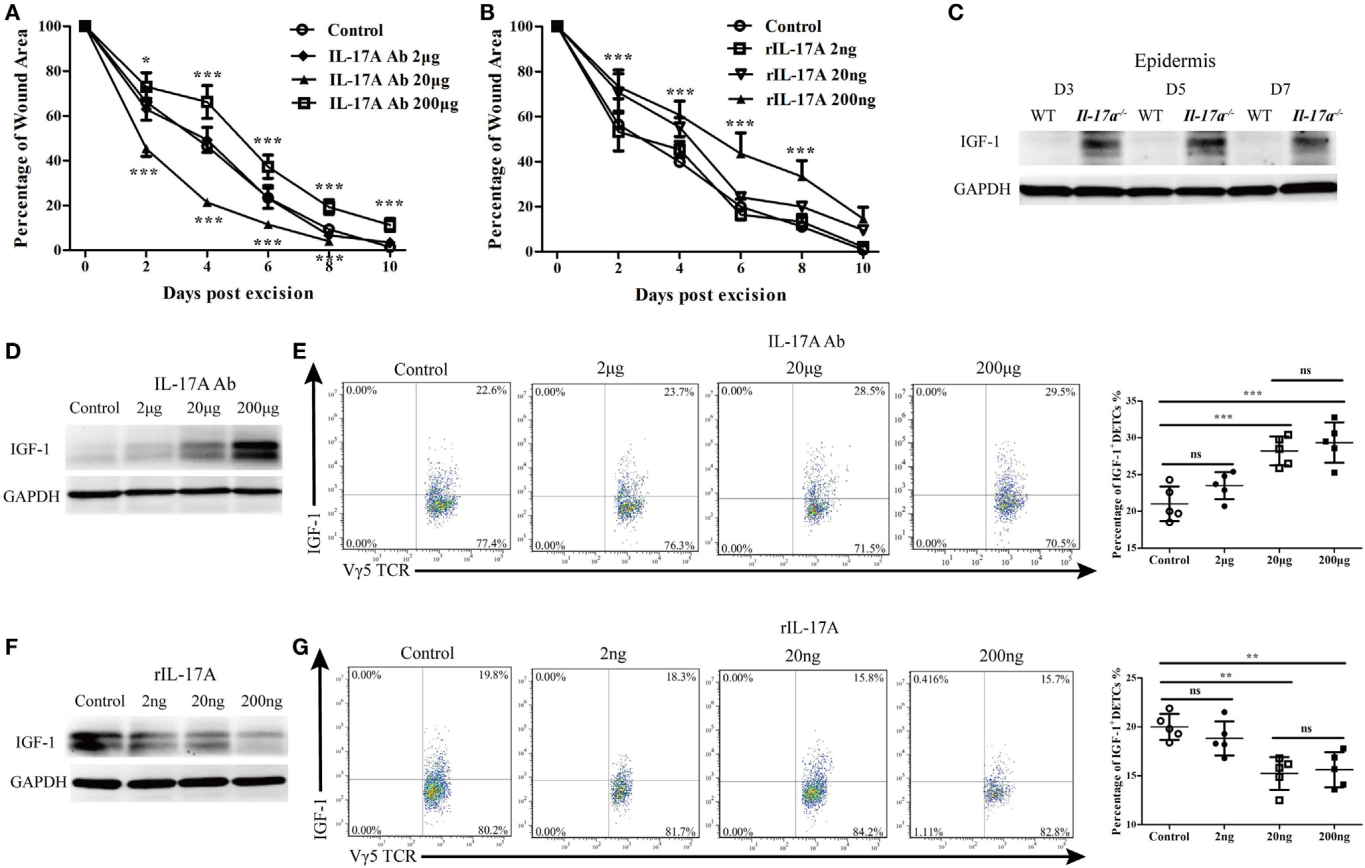
IL-17A inhibited the production of IGF-1 in epidermis around wound after skin injury. **(A)** Addition of various doses of IL-17A neutralizing Ab (0, 2, 20, and 200 μg/wound) into wound margin of WT mice on days 0, 1, and 2 after skin injury. Wound-closure kinetics was measured over time and dots in wound closure kinetics represent 10 wounds per group (2 wounds/mouse, 5 mice/group). Upper stars stand for the comparations between IL-17A Ab 200 μg and Control, and lower stars represent the comparations between IL-17A Ab 20 μg and Control. **(B)** Wound closure kinetics in WT mice treated with various doses of rIL-17A (0, 2, 20, and 200 ng/wound) on days 0, 1, and 2 after skin injury (*n* = 10 wounds/group). Stars stand for the comparations between rIL-17A 200 ng and Control. **(C)** The production of IGF-1 in epidermis around wound of WT and *IL-17a*^−/−^ mice on days 3, 5, and 7 post-excision was examined by WB. **(D,E)** Addition of various doses of IL-17A neutralizing Ab (0, 2, 20 and 200 μg/wound) into wound margin of WT mice on days 0, 1, and 2 after skin injury. Epidermis around wound was collected on day 3 post-excision. IGF-1 expression was determined by WB **(D)** and FACS **(E)**. **(F,G)** Addition of various doses of rIL-17A (0, 2, 20, and 200 ng/wound) into wound margin of WT mice on days 0, 1, and 2 after skin injury. Epidermis around wound was collected on day 3 post-excision. The production of IGF-1 was detected by WB **(F)** and FACS **(G)**. Epidermal sheets around wounds were collected from 3–7 mice with 4 wounds/mice in **(C–G)**. Data are representative of three individual experiments. Bars are shown mean ± SD. *P* value was determined by one-way ANOVA with Student’s unpaired *t*-test **(A,B)** or Bonferroni’s comparision test **(E,G)** (*P < 0.05, **P < 0.01, ***P < 0.001).

IL-17A has been demonstrated to promote keratinocyte proliferation for efficient skin wound repair (4). Consistent with previous reports, we showed that both IL-17A deficiency (Figure S2 in Supplementary Material) and blocking IL-17A with a high dose of neutralizing Ab (200 μg/wound) in wound margins leads to defective skin wound closure (IL-17A Ab 200 μg vs. control, Figure 6A). Interestingly, the addition of a moderate dose of anti-IL-17A neutralizing Ab (20 μg/wound) significantly improved skin wound repair (IL-17A Ab 20 μg vs. control, Figure 6A). Moreover, the administration of a high dose of rIL-17A (200 ng/wound) rather than low or medium dose (2 or 20 ng/wound) onto wound beds significantly retarded skin wound closure (rIL-17A 200 ng vs. control, Figure 6B). Thus, these findings indicated that the excessive IL-17A has a negative impact on skin wound repair, although IL-17A is essential for efficient wound healing.

Since IGF-1 is also required for efficient skin wound repair, we further investigated whether IL-17A affected IGF-1 production in the epidermis after wounding. As expected, *Il-17a*^−/−^ mice, exhibited a defective skin wound repair and showed a markedly enhanced IGF-1 production in the epidermis around wounds compared to WT controls (Figure 6C). The addition of various doses of anti-IL-17A neutralizing Ab onto wound beds notably increased epidermal IGF-1 (Figure 6D) and IGF-1 production in DETCs (Figure 6E) at wound areas of WT mice in a dose-dependent manner. Moreover, supplementing various doses of rIL-17A onto wound beds markedly decreased IGF-1 production in the epidermis (Figure 6F) and IGF-1 production in DETCs (Figure 6G) around wounds of WT mice in a dose-dependent manner. These results demonstrated that IL-17A was able to suppress the production of epidermal IGF-1 in DETCs after skin injury.

### IL-1β and IL-23 Directly Inhibit IGF-1 Production in DETCs to Delay Wound Healing

**Figure 7:**
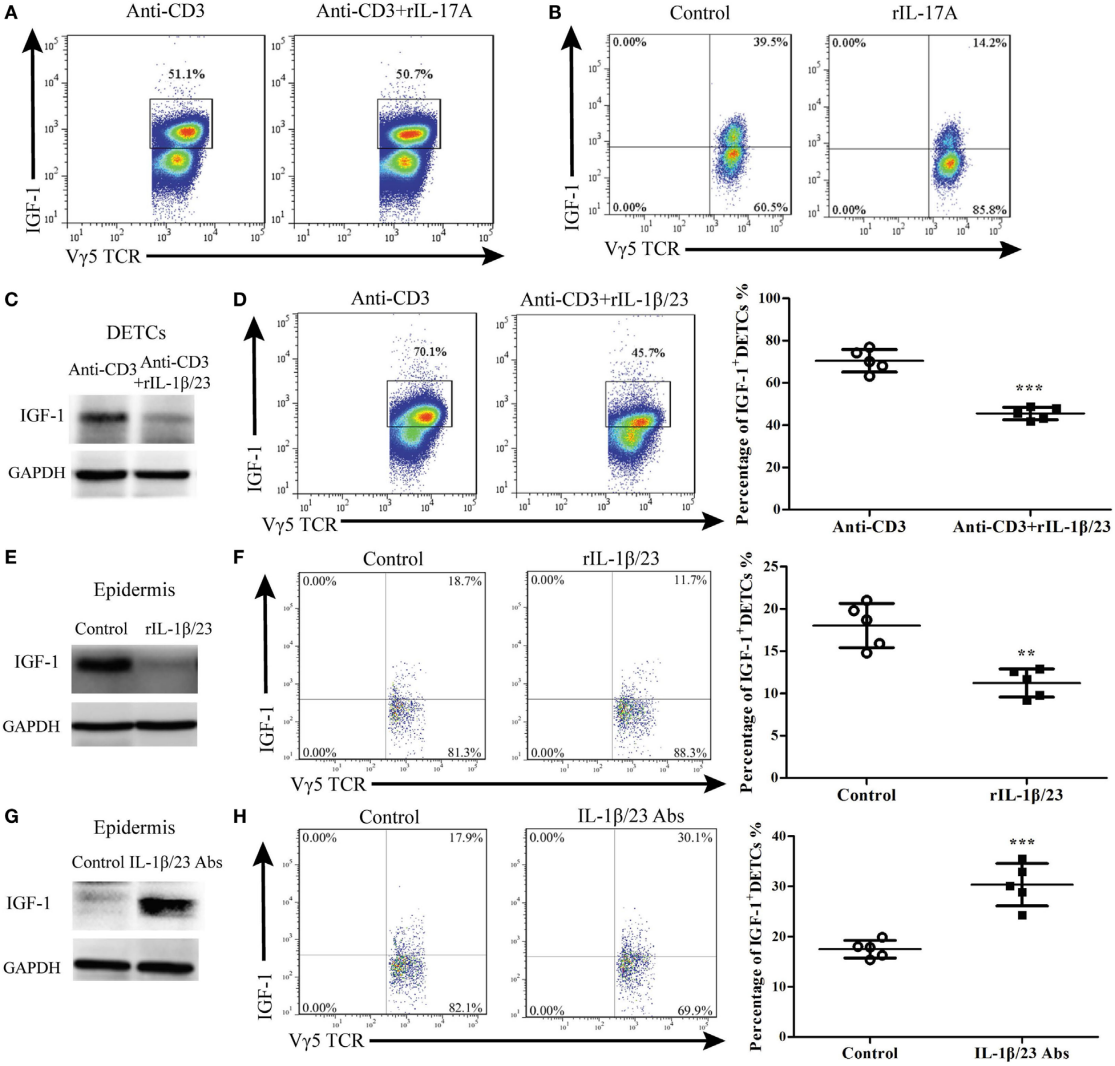
IL-1β and IL-23 directly inhibited IGF production by dendritic epidermal T cells (DETCs). **(A)** DETCs were isolated from wild-type (WT) mice and expanded with Con A stimulation for 4 weeks. The expanded DETCs (eDETCs) (purity > 95%) were rested without Con A for 2 weeks before further analysis. eDETCs were stimulated for 6 h with anti-CD3ε (5 μg/ml) either alone or combined with rIL-17 (100 ng/ml) in the presence of brefeldin A (BFA) (100 ng/ml). IGF-1 productions by eDETCs were analyzed by FACS. **(B)** eDETCs were co-cultured with keratinocytes (1:1, 1 × 10^6^/ml) and stimulated by rIL-17 in presence of anti-CD3ε for 6 h. IGF-1 expression in eDETCs was detected by FACS. **(C,D)** eDETCs were stimulated with anti-CD3ε either alone (Anti-CD3) or combined with rIL-1β (100 ng/ml) plus rIL-23 (100 ng/ml) (anti-CD3 + rIL-1β/23) in the presence of BFA for 6 h. The expression of IGF-1 in eDETCs was detected by WB **(C)** and FACS **(D)**. **(E,F)** Age- and sex-matched WT mice were treated with rIL-1β (20 ng/wound) plus rIL-23 (20 ng/wound) on days 0, 1, and 2 after wounding. Epidermis around wound was collected from these animals on day 3 post-excision. The productions of IGF-1 in epidermis around wound were detected by WB **(E)** and FACS **(F)**. **(G,H)** Age- and sex-matched WT mice were treated with IL-1β neutralizing Ab (20 μg/wound) plus IL-23 neutralizing Ab (20 μg/wound) on days 0, 1, and 2 after wounding. Animals with IL-1β isotype Ab (armenian hamster IgG) plus IL-23 isotype Ab (rat IgG2a) treatment were used as control. Epidermis around wound was collected from these animals on day 3 post-excision. IGF expression was determined by WB **(G)** and FACS **(H)**. Epidermal sheets around wounds were gained from 3 to 5 mice with 4 wounds/mice in **(E–H)**. Data represent three individual experiments. Bars are shown mean ± SD. *P* value was assessed by Student’s unpaired *t*-test **(D,F,H)** (**p < 0.01, ***p < 0.001).

To investigate the direct effect of IL-17A on IGF-1 production in DETCs, we isolated, purified, and expanded DETCs *in vitro* (eDETCs) and co-cultured them with rIL-17A. The results showed that rIL-17A failed to inhibit the production of IGF-1 in eDETCs (Figure 7A). Although we observed that IL-17A could inhibit the pro-healing function of DETCs *in vivo*, DETCs did not directly respond to IL-17A. However, in the co-culture system of eDETCs and primary keratinocytes isolated from newborn C57 mice, rIL-17A notably suppressed IGF-1 production in eDETCs (Figure 7B), suggesting that IL-17A retarded IGF-1 production in DETCs *via* epidermal cells.

**Figure 8:**
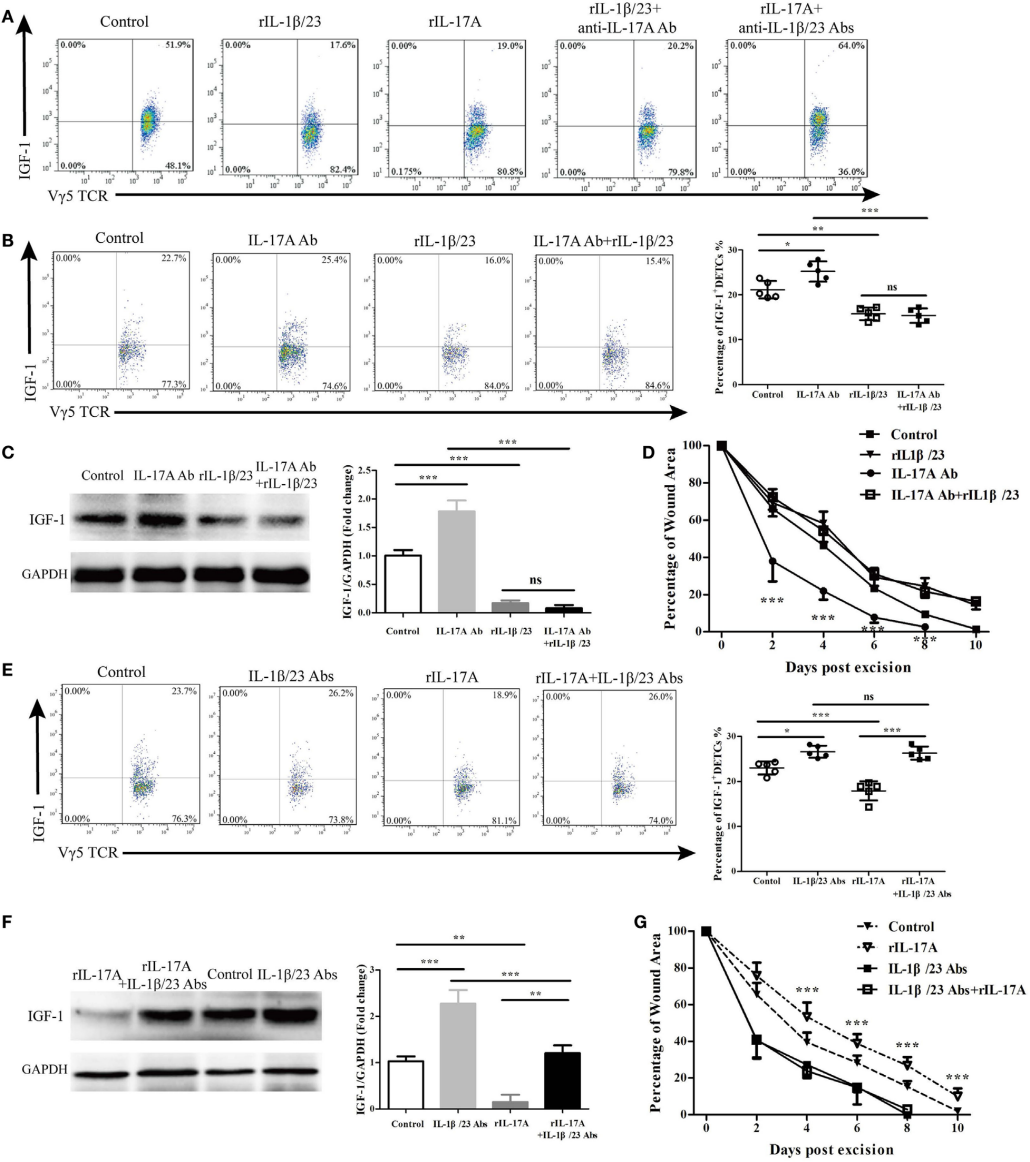
IL-17A *via* IL-1β and IL-23 reduced IGF-1 production by dendritic epidermal T cells (DETCs). **(A)** eDETCs co-cultured with keratinocytes (1:1, 1 × 10^6^/ml) in presence of rIL-1β/rIL-23 (100 ng/ml), rIL-17A (100 ng/ml), rIL-1β/rIL-23 plus anti-IL-17A Ab (10 μg/ml), or rIL-17A plus anti-IL-1β/anti-IL-23 Abs (10 μg/ml) for 6 h. IGF-1 production by eDETCs was detected by FACS. **(B–D)** Age- and sex-matched wild-type (WT) mice were treated with IL-17A neutralizing Ab (20 μg/wound), rIL-1β/rIL-23 (20 ng/wound), or IL-17A neutralizing Ab plus rIL-1β/rIL-23 (20 ng/wound) for continuously 3 days after wounding. IGF-1 expression in DETCs **(B)** and epidermal sheet **(C)** around wound was detected by FACS and WB, respectively, on day 3 post-excision (4 wounds/mice, 3 mice/group). **(D)** Wound closure kinetics over time was shown (*n* = 10 wounds/group). Stars mark the comparations between IL-17A Ab and IL-17A Ab + rIL-1β/rIL-23. **(E–G)** Age- and sex-matched WT mice were treated with IL-1β/IL-23 neutralizing Abs (20 μg/wound), rIL-17A (20 ng/wound), rIL-17A plus IL-1β/IL-23 neutralizing Abs for continuously 3 days after wounding. IGF-1 expression in DETCs **(E)** and epidermis **(F)** around wound was analyzed by FACS and WB on day 3 post-excision (4 wounds/mice, 3 mice/group). **(G)** Wound closure kinetics over time was shown (*n* = 10 wounds/group). Stars represent the differences between rIL-17A and rIL-17A + IL-1β/IL-23 Abs. Data represent three individual experiments. Bars are shown mean ± SD. *P* value was assessed by One-way ANOVA with Bonferroni’s comparison test (*P < 0.05, **P < 0.01, ***P < 0.001).

The IL-1β/23-IL-17A axis plays a crucial role in inflammation (9, 19–22). It has been shown that the production of IL-17A by DETCs could be markedly enhanced in response to IL-1β and IL-23 upon TCR stimulation (4, 22). We investigated whether IL-1β and IL-23 could directly affect IGF-1 production in DETCs. The results showed that rIL-1β and rIL-23 markedly inhibited IGF-1 production by eDETCs *in vitro* (WB, Figure 7C; FACS, Figure 7D). Furthermore, application of rIL-1β and rIL-23 onto wound beds of WT mice markedly decreased IGF-1 production in the epidermis (Figure 7E) and DETCs (Figure 7F) around wounds and subsequently delayed skin wound repair (Figure 8D). The supplement of IL-1β/IL-23 neutralizing antibodies onto wound beds of WT mice notably enhanced IGF-1 production in the epidermis (Figure 7G) and DETCs (Figure 7H) around wounds, followed by improved skin wound closure (Figure 8G). These results suggest that IL-1β and IL-23 could directly affect IGF-1 production in DETCs.

### IL-1β and IL-23 Are Intermediaries between IL-17A and IGF-1 in the Epidermis around Wounds

In the co-culture system of eDETCs and primary keratinocytes, rIL-1β and rIL-23 reduced IGF-1 expression by eDETCs *in vitro* (Figure 8A). rIL-17 also inhibited the production of IGF-1 by eDETCS in the co-culture system (Figure 8A), while blocking IL-17A together with the application of rIL-1β and rIL-23 failed to rescue the reduction of IGF-1 in eDETCs *in vitro* (Figure 8A). However, blocking IL-1β and IL-23 with neutralizing antibodies together with the addition of rIL-17A almost eliminated the reduction of IGF-1 in eDETCs mediated by IL-17A *in vitro* (Figure 8A). *In vivo*, the production of IGF-1 in eDETCs was enhanced by blocking IL-17A (20 μg/wound) on wound beds of WT mice, while the expression of IGF-1 was decreased when adding rIL-1β plus rIL-23. Blocking IL-17A could not rescue the declined production of IGF-1 (Figure 8B). The epidermal expression of IGF-1 around wounds was assessed by western blot and showed a similar result to FACS (Figure 8C). Wound repair was significantly promoted by blocking IL-17A alone compared to the application of rIL-1β and rIL-23 together with blocking of IL-17A (IL-17A Ab vs. IL-17A Ab + rIL-1β/23, Figure 8D). Supplying rIL-17A to wound margins could weaken IGF-1 production in eDETCs (Figure 8E) and the epidermis around wounds (Figure 8F), whereas the addition of rIL-17 with IL-1β, and IL-23 neutralization failed to display this effect (Figures 8E,F). Compared to the addition of rIL-17 alone, rIL-17 supplied with IL-1β combined with IL-23 neutralization completely reversed wound healing kinetics to control levels (rIL-17A vs. rIL-17A + IL-1β/IL-23 Abs, Figure 8G). These findings indicated that IL-1β and IL-23 acted as intermediaries between IL-17A and IGF-1 to impact wound closure.

### IL-17A and IL-1β/IL-23 Form a Positive Feedback in the Epidermis around Wounds

**Figure 9:**
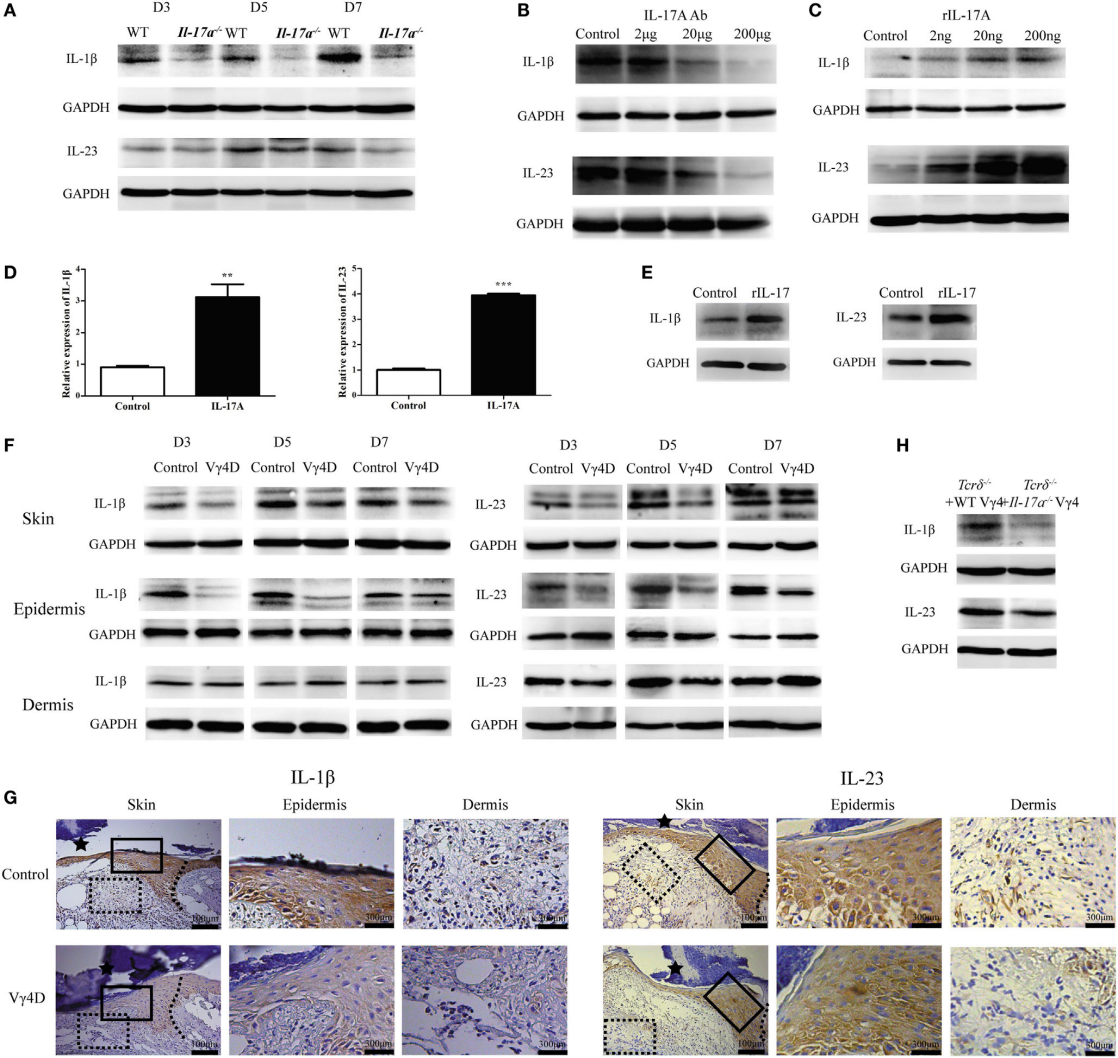
Vγ4 T cell-derived IL-17A enhanced the production of IL-1β and IL-23 in epidermis around wound. **(A)** The expression of epidermal IL-1β and IL-23 around wound of wild-type (WT) and *Il-17a*^−/−^ mice was analyzed by WB on days 3, 5, and 7 post-excision (*n* = 3/group). **(B)** Age- and sex-matched WT mice received various doses of IL-17A neutralizing Ab treatment (doses of 0, 2, 20, and 200 μg/wound) in the wound margin on days 0, 1, and 2 post excision. WB analysis of IL-1β and IL-23 expression in epidermal wound margin on day 3 post excision (*n* = 3/group). **(C)** WT mice were injected with various doses of rIL-17A treatment (doses of 0, 2, 20, and 200 ng/wound) in the wound margin for continuous 3 days following excision. IL-1β and IL-23 expression in epidermal wound margin was analyzed by WB on day 3 post excision (*n* = 3/group). **(D,E)** Fresh isolated primary keratinocytes cultured with IL-17A (100 ng/ml) for 24 h. The expression of IL-1β and IL-23 was measured by qPT-PCR **(D)** and WB **(E)**. **(F,G)** The expression of IL-1β and IL-23 in whole skin, epidermis, and dermis around wound of Vγ4D and WT control mice was assessed by WB on days 3, 5, and 7 post-excision **(F)** and IHC **(G)** on day 3 post-excision. **(H)** WT or *Il-17a*^−/−^ Vγ4 T cells were added onto wound bed of *Tcrδ*^−/−^ mice upon wounding (*n* = 4/group). The production of IL-1β and IL-23 in epidermis at wound edge was analyzed by WB on day 3 post-excision. Data represent three independent experiments using the indicated number of mice in brackets for each group or mouse strain. *P* value was assessed by Student’s unpaired *t*-test (**P < 0.01, ***P < 0.001).

After showing that IL-1β and IL-23 were required for IL-17A to inhibit IGF-1 production in DETCs, we wanted to determine the precise role of IL-17A in epidermal IL-1β/IL-23 production *in vivo* and *in vitro*. The *in vivo* production of both IL-1β and IL-23 in the epidermis around wounds was markedly reduced in *Il-17a*^−/−^ mice compared to WT controls (Figure 9A). IL-17A neutralization attenuated IL-1β and IL-23 production in the epidermis, as increasing doses of IL-17 neutralization led to the reduced expression of IL-1β and IL-23 (Figure 9B). Moreover, the administration of rIL-17A enhanced the production of epidermal IL-1β and IL-23 in a dose-dependent manner (Figure 9C). rIL-17A notably enhanced the *in vitro* production of IL-1β and IL-23 by primary keratinocytes at both the mRNA (Figure 9D) and protein (Figure 9E) levels, suggesting that IL-17A is an important factor to enhance epidermal IL1/23 production in the epidermis around wounds after skin injury.

Furthermore, we investigated the effect of Vγ4 T cells on epidermal IL-1β and IL-23 production. Vγ4D markedly reduced IL-1β and IL-23 production in the epidermis rather than the dermis around wounds in WT mice (WB, Figure 9F; IHC, Figure 9G). Compared to *Il-17a*^−/−^ Vγ4 T cells, supplementing WT Vγ4 T cells onto wound beds of *Tcrδ*^−/−^ mice markedly improved epidermal IL-1β and IL-23 production around wounds (Figure 9H). These results indicated that IL-17A was not only stimulated by IL-1β and IL-23 but also promoted IL-1β/IL-23 production in the epidermis to form a positive feedback loop after skin injury.

### IL-1β Rather Than IL-23 Plays a Crucial Role in the Regulation of IGF-1 Production by DETCs in an NF-κB-Dependent Manner

**Figure 10:**
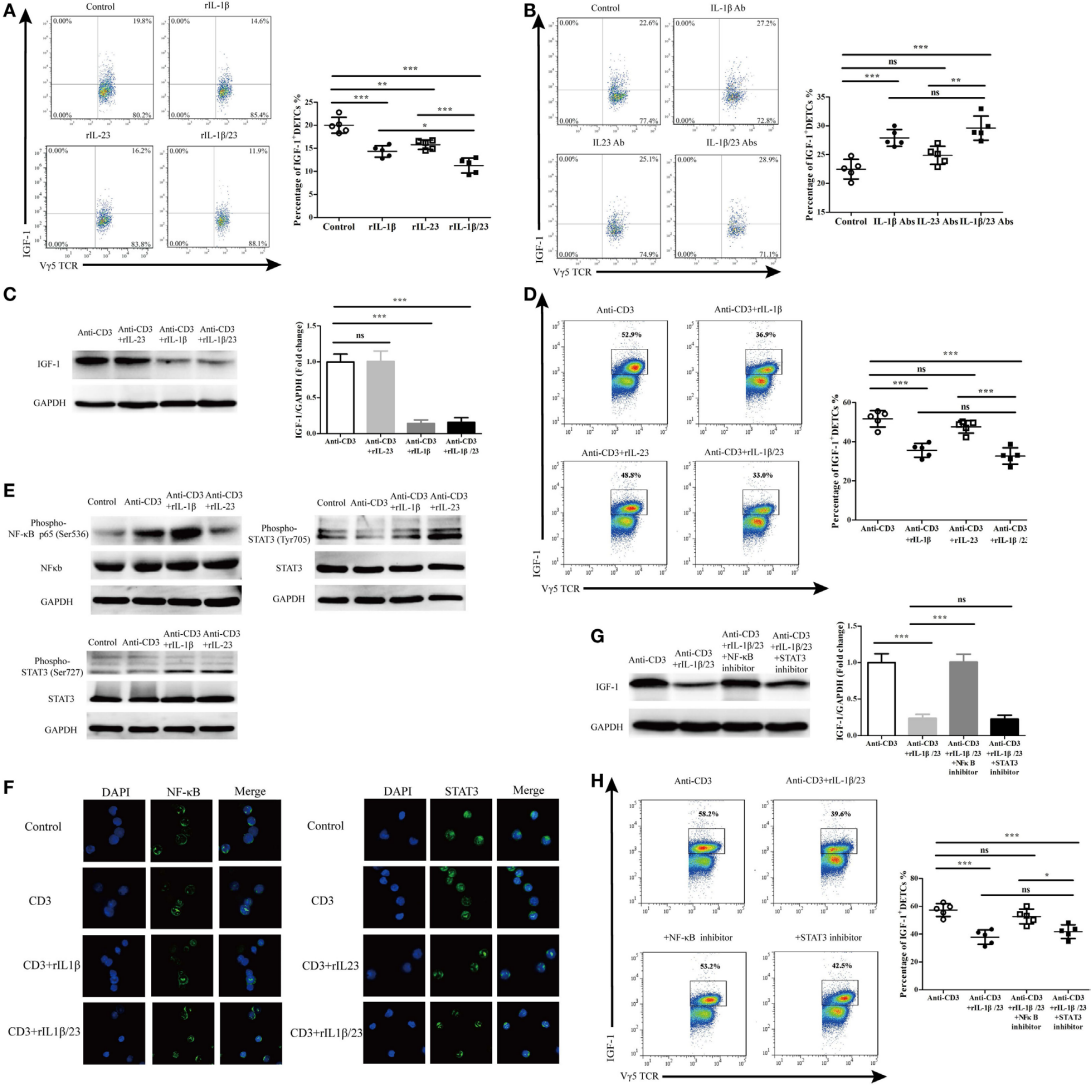
IL-1β rather than IL-23 significantly decreased IGF-1 production by dendritic epidermal T cells (DETCs) in NF-κB-dependent manner. **(A)** FACS analysis of IGF-1 production in DETCs around wound of wild-type (WT) mice with rIL-1β, rIL-23, or rIL-1β plus rIL-23 treatment in the wound margin for continuous 3 days following excision (4 wounds/mice, 3 mice/group). **(B)** FACS analysis of IGF-1 production by DETCs around wound of WT mice with IL-1β-neutralizing Ab, IL-23-neutralizing Ab, or IL-1β-neutralizing Ab plus IL-23-neutralizing Ab treatment in the wound margin for continuous 3 days following excision (4 wounds/mice, 3 mice/group). **(C,D)** eDETCs were rested for 2 weeks without ConA and then co-cultured with rIL-1β, rIL-23, or rIL-1β plus rIL-23 upon anti-CD3ε stimulation for 6 h. The production of IGF-1 in eDETCs was measured by WB **(C)** and FACS **(D)**. **(E,F)** eDETCs co-cultured with rIL-1β or rIL-23 upon anti-CD3ε stimulation for 30 min. **(E)** Expression of phospho-NF-κB p65 (Ser536), total NF-κB p65, phosphor-STAT3 (Ser727), phosphor-STAT3 (Ser705), and total STAT3 was measured by WB. **(F)** Confocal immunofluorescence was used to check nucleus translocation of NF-κB p65 and STAT3. **(G,H)** eDETCs co-cultured with rIL-1β plus rIL-23, rIL-1β/rIL-23 plus NF-κB inhibitor (PDTC), or rIL-1β/rIL-23 plus STAT3 inhibitor (S3I-201) upon anti-CD3ε stimulation for 6 h. WB **(G)** and FACS **(H)** were performed to analyze IGF-1 expression by eDETCs. Data are representative of three individual experiments. Bars are shown mean ± SD. Data are representative of at least three independent experiments. Error bars represent mean ± SD. One-way ANOVA with Bonferroni comparison test was used to calculated *P* value (*P < 0.05, **P < 0.01, ***P < 0.001).

We further investigated the precise roles of IL-1β and IL-23 in inhibiting IGF-1 production by DETCs *in vivo* and *in vitro*. The application of rIL-1β, rIL-23, or rIL-1β plus rIL-23 onto wound beds markedly decreased IGF-1 production by DETCs around wounds after skin injury (Figure 10A). Interestingly, the IGF-1 production by DETCs was significantly reduced in mice with rIL-1β plus rIL-23 treatment compared to those with rIL-1β or rIL-23 treatment alone (Figure 10A). Moreover, the administration of IL-1β neutralizing Ab alone or IL-1β plus IL-23 neutralizing Abs together onto wound beds notably increased IGF-1 production by DETCs around wounds after skin injury. However, IL-23-neutralizing Ab alone did not exhibit a similar effect (Figure 10B). Similar to the *in vivo* results, the addition of rIL-1β alone or rIL-1β plus rIL-23 rather than rIL-23 alone could remarkably inhibit IGF-1 production by eDETCs *in vitro* (WB, Figure 10C; FACS, Figure 10D). These findings suggested that IL-1β suppressed IGF-1 production in DETCs, and IL-23 could only enhance the inhibition mediated by IL-1β.

We next examined the IL-1β/IL-23 downstream pathways such as phosphorylated NF-κb and STAT3 (24–27). As expected, IL-1β and IL-23 markedly enhanced the phosphorylation (Figure 10E) and the translocation of NF-κb and STAT3 from the cytoplasm to the nucleus (Figure 10F) in eDETCs upon anti-CD3e stimulation *in vitro*. Moreover, blocking NF-κb signaling rather than STAT3 remarkably increased the expression of IGF-1 by eDETCs upon anti-CD3e stimulation *in vitro* (WB, Figure 10G; FACS, Figure 10H). Thus, we concluded that NF-κb signaling may play a more critical role in the IL-1β/IL-23 regulation of IGF-1 production in DETCs.

## Discussion

Wound healing is a complex process divided into four phases of hemostasis, inflammation, proliferation, and remodeling (28). Murine γδ T cells are the major T lymphocyte subsets that are distributed in skin tissue and engage in inflammation and re-epithelialization in wound repair (7, 28, 29). Skin γδ T cells consist of several subsets with distinctive functions. DETCs are the major source of epidermal IGF-1 that promote re-epithelialization (7), and Vγ4 T cells provide IL-17A in the early stages of skin inflammation (1, 15). It has been demonstrated that both impaired and excessive inflammation suppresses wound healing (4, 17). However, whether inflammation affects wound healing and how skin γδ T cell subsets are involved in this process remain unknown. In this study, we demonstrated that Vγ4 T cells provided IL-17A in the epidermis around wounds at early stages after skin injury, and IL-17A inhibited IGF-1 production by DETCs around wounds to delay wound healing. It is noteworthy that we used anti-Vγ4 TCR Ab (UC3-10A6) purchased from BioXcell to deplete Vγ4 T cells according to Hartwig et al. and Suryawanshi et al. (30, 31). However, Koenecke et al. identified that *in vivo* treatment with both GL3 and UC7-13D5 antibodies against TCR did not deplete γδ T cells but instead caused TCR internalization (32). Therefore, we analyzed the percentages of Vγ1 T cells and Vγ4 T cells in the spleen or lymph nodes 5 weeks after Vγ1 or Vγ4 T cell depletion and confirmed that the efficacy of Vγ1 or Vγ4 T cell depletion was excellent and could last for at least 5 weeks (data not shown). Furthermore, we have examined Vγ4 T cell percentages in the dermis from 3 days to 28 weeks after Vγ4 T cell depletion and confirmed that the efficacy of Vγ4 T cell depletion was excellent and could last for at least 3 months (data not shown). These results at least partially confirm that InVivoMAb treatment can deplete Vγ4 T cells *in vivo* because the downregulation of Vγ4 TCR complexes is impossible to recover after some time. But, we used the same clone Ab (UC3-10A6) to deplete and detect Vγ4 T cells, which cannot distinguish cells that have had their TCR downregulated from depletion. Therefore, we could still not exclude the possibility that Ab treatment does not deplete γδ T cells but rather reduces their TCR complexes on the cell surface.

Dendritic epidermal T cell-derived IGF-1 is necessary for epithelial homeostasis and protects keratinocytes in injured areas from apoptosis to aid re-epithelialization (7). DETCs around wounds provide IL-17A to assist in wound healing by inducing epidermal keratinocytes to express antimicrobial peptides and proteins such as β-defensin 3, S100A8, and RegIIIγ. Therefore, DETC-derived IL-17A is regarded as essential to reestablish the antimicrobial skin barrier after skin injury (4). *Tcrδ*^−/−^ mice exhibit wound healing defects, which are rescued by the reconstitution of DETCs or addition of recombinant IGF-1 (7, 28). Similarly, wound-healing defects in *Il-17a*^−/−^ animals could be restored by the addition of rIL-17A, IL-17A-producing DETCs (4), or IL-17A-producing Vγ4 T cells (data not shown). In addition, our previous studies demonstrated that both IGF-1 and IL-17A production in diabetic wounds were impaired, and the application of DETCs or IL-17A-positive Vγ4 T cells could improve the defects of diabetic wound healing (16, 33). Although IL-17A is required for efficient skin wound healing, excessive IL-17A displayed a harmful impact on skin wound closure (34). In this study, we revealed that epidermal IGF-1 production, which is required for efficient wound healing, could be downregulated by IL-17A after skin injury. Deficiency or blockage of IL-17A enhanced IGF-1 production in the epidermis and DETCs around wounds *in vivo*, whereas the addition of rIL-17A attenuated this effect. In the co-culture system of keratinocytes and eDETCs, blocking IL-17A with neutralizing Ab increased IGF-1 production by eDETCs *in vitro*, whereas the addition of rIL-17A resulted in the opposite outcome. Furthermore, we determined that Vγ4 T cell-derived IL-17A inhibited IGF-1 production by DETCs to delay wound repair. Vγ4 T cell depletion enhanced epidermal IGF-1 production and promoted wound healing. Addition of WT rather than *Il-17a*^−/−^ Vγ4 T cells onto wound beds of *Tcrδ*^−/−^ mice significantly attenuated epidermal IGF-1 production and wound healing, which was facilitated by supplementing WT DETCs. Thus, these findings suggested a previously unrecognized role of IL-17A to downregulate epidermal IGF-1 production, which was critical for Vγ4 T cells to impair wound healing.

Vγ4 T cells provide a major source of IL-17A to participate in skin autoimmune diseases (1, 19). In this study, we further confirmed that Vγ4 T cells provided major source of IL-17A in the epidermis at early stages of wounding. Approximately half of the epidermal IL-17A-positive cells are Vγ4 T cells on day 3 after wounding. Depletion of Vγ4 T cells dramatically decreased epidermal IL-17A production in WT mice, and the addition of freshly isolated Vγ4 T cells onto wound beds significantly enhanced epidermal IL-17A production in *Tcrδ*^−/−^ animals. It is worth mentioning that DETCs have also been reported as a source of epidermal IL-17A for efficient wound healing (4, 7). Indeed, more than 15% of eDETCs, which expanded *in vitro* with ConA for 2–4 weeks, expressed IL-17A upon anti-CD3 stimulation (Figure S3 in Supplementary Material). However, few IL-17A-positive DETCs were detected in the epidermis around wounds after skin injury, and the addition of freshly isolated DETCs onto wound beds failed to significantly enhance epidermal IL-17A production in *Tcrδ*^−/−^ animals. Therefore, Vγ4 T cells rather than DETCs provided a major source of IL-17A in the epidermis after wounding.

The IL-1β/IL-23-IL-17A axis is critical for the initiation and amplification of inflammatory responses (9, 19–22). Interestingly, our previous results suggested that IL-17A might also act upstream to enhance epidermal IL-1β/IL-23 production in a skin graft transplantation model (18). Here, we defined that IL-17A and IL-1β/IL-23 form a positive feedback loop in the epidermis around wounds to amplify local inflammation after skin injury. The epidermal IL-1β and IL-23 production at wound edges was weakened by a deficiency or blockage of IL-17A but enhanced by the addition of rIL-17A. Moreover, we revealed that Vγ4 T cell-derived IL-17A impacted epidermal IL-1β/IL-23 production after wounding. Depletion of Vγ4 T cells resulted in decreased epidermal IL-1β/IL-23 production, and supplementing WT rather than *Il-17a*^−/−^ Vγ4 T cells onto wound beds promoted epidermal IL-1β/IL-23 production in *Tcrδ*^−/−^ mice. In addition, IL-17A-producing γδ T cells express high levels of CCR6 on their surface and are recruited by CCL20 to migrate toward inflammatory sites (35). We observed that CCL20 neutralization dramatically decreased the infiltration of Vγ4 T cells into the epidermis around wounds and reduced epidermal IL-17A production. Together, IL-1β/IL-23 and CCL20 are thought to amplify epidermal IL-17A production by Vγ4 T cells and thereby exacerbate local inflammation.

Both Vγ4 T cells and DETCs have been shown to produce IL-17A in the presence of IL-1β and IL-23 (4, 19, 22). Here, we identified that IL-1β/IL-23 rather than IL-17A directly inhibited IGF-1 in DETCs. Furthermore, we revealed that IL-1β and IL-23 were crucial for IL-17A to inhibit IGF-1 production by DETCs. In the co-culture system of keratinocytes and eDETCs, blocking IL-1β/IL-23 totally reversed the reduction of IGF-1 in eDETCs caused by rIL-17A, whereas blocking IL-17A failed to rescue the reduction of IGF-1 by eDETCs mediated by rIL-1β/rIL-23. Blocking IL-1β/IL-23 consistently eliminated the inhibition of epidermal IGF-1 and the delay of wound healing, which were caused by rIL-17A *in vivo*, whereas blocking IL-17A failed to rescue the reduction of epidermal IGF-1 and the delay of wound healing, which were mediated by rIL-1β/rIL-23 *in vivo*.

Activated DETCs not only secrete IGF-1 but also produce IL-17A in the presence of IL-1β and IL-23 (4, 7). Here, we found a negative correlation between IGF-1 and IL-17A production in DETCs, which was closely regulated by IL-1β rather than IL-23. The addition of rIL-1β together with rIL-23 markedly reduced IGF-1 but enhanced IL-17A production by eDETCs *in vitro* (Figure S3 in Supplementary Material). Moreover, IL-1β rather than IL-23 has been shown to regulate IGF-1 and IL-17A production by DETCs in an NF-κb-dependent manner. Applying rIL-1β rather than rIL-23 had similar effects on eDETCs to rIL-1β together with rIL-23 (Figure S3 in Supplementary Material). Blocking NF-κb rather than STAT3 signaling with drugs completely eliminated the reduction of IGF-1 and enhancement of IL-17A by eDETCs mediated by rIL-1β/rIL-23 *in vitro* (Figure S3 in Supplementary Material). In addition, blocking IL-1β rather than IL-23 notably enhanced IGF-1 production by DETCs around wounds *in vivo*. However, rIL-1β and rIL-23 have exhibited a limited ability to enhance IL-17A production by DETCs that were freshly isolated from intact or wounded skin (Figure S4 in Supplementary Material). This suggests that, with long-term strong stimulation, DETCs may change to pro-inflammation cells to provide IL-17A in refractory wounds. This issue needs to be deeply investigated in the future.

TCR signaling is required for the survival and activation of T lymphocytes. DETCs rely on the recognition of TCR Vγ5 to unidentified ligands on epidermal cells under steady and stressed states (36). Interestingly, part of Vγ4 T cells, isolated from spleen/lymph nodes and expanded with anti-CD3 or anti-Vγ4 Ab *in vitro*, produces IL-17A upon PMA plus ionomycin or anti-CD3 re-stimulation (data not shown). Whether epidermal-infiltrating Vγ4 T cells that produce IL-17A are associated with some ligands expressed on stressed epidermal cells is still unclear. With the exception of TCR signaling, NKG2D has been demonstrated to activate γδ T cells (37). Rae1 and H60, ligands of NKG2D, are expressed on the surface of keratinocytes and could be markedly enhanced by inflammatory mediators (38, 39). Our data showed that Vγ4D significantly improved the expression of NKG2D on DETCs around wounds. Whether and how NKG2D regulates IGF-1 production by DETCs need to be investigated. Moreover, BTLA has been reported as a negative regulatory signal for γδ T cell activation until now (40). Whether and how BTLA affects the pro-inflammatory function of Vγ4 T cells and pro-healing function of DETCs need to be clarified in the future.

Our data established a mechanistic link between Vγ4 T cell-derived IL-17A, epidermal IL-1β/IL-23, DETC-derived IGF-1, and wound repair in the skin. The functional balance between Vγ4 T cells and DETCs resulted in efficient skin wound closure. Since that microbiological status at the wound area is very important for the outcome of skin wound healing, further investigation is needed into how Vγ4 T cell-mediated inflammation affects the defense from pathogen invasion after wound after skin injury.

## Experimental Procedures

### Mice

C57BL/6 WT and *Il-17a*^−/−^ mice were obtained from the Animal Center of the Third Military Medical University (Chongqing, CN, USA). TCRδ-GFP, *Tcrδ*^−/−^ and *Ifn-γ*^−/−^ mice on C57BL/6 background were purchased from Jackson Laboratory (Bar Harbor, ME, USA). Sex-matched mice aged 6–8 weeks were used for all experiments. All experiments were performed under specific pathogen-free conditions in conformity with ethical guidelines and approved by the Animal Ethics Committee of the Third Military Medical University.

### Preparation of Full-Thickness Excision Wound

Excision wounds were cut down to the musculus panniculus carnosus with a diameter of 6-mm sterile punch. For wound model without contraction, glue the biological membrane (negative pressure wound therapy kit, China) with the adhesive dressings immediately onto the surface of the wound before contraction and single-house the mice. Wound area was recorded daily by macroscopic digital photographs and estimated at each time point relative to day 0 until completely closure.

### Mouse Models of Vγ1/Vγ4 T Cell Depletion, IL-17A/IFN-γ/IL-1β/IL-23/CCL20 Neutralization and rIGF-1/rIL-17A/rIL-1β/rIL-23 Addition

Mouse model of Vγ1D was implemented by intraperitoneal injection of 200 μg anti-Vγ1 Ab (clone 2.11; BioXcell, USA) 3 days before wound excision. Vγ4D was generated by anti-Vγ4 Ab (clone UC3-10A6; BioXcell, USA). Control mice were intraperitoneally administrated isotype control (armenian hamster IgG) (BioXcell, USA). Neutralization mouse models were conducted by injecting subcutaneously into wound margin 0, 1, and 2 days following excision with 20 μg/wound of anti-IGF-1 Ab (R&D Systems, Minneapolis, MN, USA), anti-IL-17A Ab (clone 17F3; BioXcell, USA), anti-IFN-γ Ab (clone XMG1.2; BioXcell, USA), anti-IL-1β Ab (clone B122; BioXcell, USA), anti-IL-23 Ab (clone BE0051; BioXcell, USA), anti-CCL20 Ab (clone 114906; R&D Systems, USA), respectively. IL-17A neutralization of low and high doses was performed by 2 and 200 μg/wound of anti-IL-17A Ab, respectively. Isotype control mice received equivalent doses of isotype Abs subcutaneously. Isotype control Abs of IGF-1, IL-17A, IL-1β, IL-23, CCL20 are poly goat IgG, mouse IgG1, armenian hamster IgG, rat IgG2, and rat IgG1 (BioXcell), respectively. Mouse models of recombinant cytokine addition were generated by subcutaneously injecting rIGF-1/rIL-17A/rIL-1β/rIL-23 (R&D Systems, Minneapolis, MN, USA) with the doses of 2, 20, 200 ng/wound, respectively, in the wound margin. Control mice were administrated with sterile PBS.

### Preparation of WT, *Il-17a*^−/−^, and *Ifn-γ*^−/−^ Vγ4 T Cells

Vγ4 T cells were isolated from lymph node cells and splenocytes of C57BL/6 WT, *Il-17a*^−/−^, and *Ifn-γ*^−/−^ mice by the EasySep positive cell isolation systems (PE labeled anti-Vγ4 Ab; BD, USA; EasySep mouse PE positive selection kit; StemCell Technologies, Canada). The purity of isolated Vγ4 T cells was identified by FACS analysis. 1 × 10^4^ cells/wound of WT, *Il-17a*^−/−^, and *Ifn-γ*^−/−^ Vγ4 T cells were placed onto the wound bed immediately following excision.

### Isolation of Epidermal Sheets and Single-Cell Suspensions

Skin that is less than 5 mm away from wound edge was regarded as wound margin. Each group of epidermis tissue around wound was collected from 3–7 mice (4 wounds/mouse) for further analysis. Subcutaneous tissue of skin was wiped off and then skin was cut into small pieces of 0.5 × 0.5 cm^2^. Epidermal sheets were torn off carefully from wound margins after incubated with 5 mg/ml dispase II at 37°C for 2 h. Epidermal single-cell suspensions were isolated by dissociating epidermal sheets with 0.3% Trypsin/GNK solution for 30 min at 37°C, then washed by PBS and filtered through a 70-μm strainer. Epidermal cells were cultured in the concentration of 5 × 10^5^–1 × 10^6^/ml with 10% FBS 1640 medium. Primary keratinocytes were isolated from skin of newborn mice in the same method as above. According to the manufacturer’s instructions (Miltenyi, GER), CD11c^+^ cells were removed from primary keratinocytes. The purity of isolated keratinocyte population was >95%.

### Preparation of DETCs

After epidermal single-cell suspension was prepared, lympholyte-M (Cedarlane Laboratories, Canada) was used to enrich DETCs (purity = 15–20%). For reconstitution assays, DETCs were further purified by the EasySep positive cell isolation systems (PE labeled anti-TCRδ Ab; BD, USA; EasySep mouse PE positive selection kit; StemCell Technologies, Canada). The purity of isolated DETCs was identified by FACS analysis (purity > 90%). Then, DETCs were transferred to wound bed immediately following excision (5 × 10^4^ cells/wound). For *in vitro* assays, DETC population was expanded in 48-well plates (1–2 × 10^6^/ml) with RPMI containing 10% FBS, 1 μg/ml Con A (Sigma-Aldrich, Germany), 10 ng/ml mouse rIL-2, 1 mM Na Pyruvate (Sigma-Aldrich, Germany), 2 mM glutamine, 25 mM HEPES, 50 μM 2-ME (Biosharp, CN, USA), 100 M non-essential amino acids (Gibco, USA), 100 μg streptomycin and 100 U penicillin for 2–4 weeks. The purity of expanded DETCs (eDETCs) was > 95% by means of FACS analysis. eDETCs were rested without ConA for 2 weeks before used for further experiments.

### Flow Cytometry Analysis and Intracellular Cytokine Staining

The following fluorochrome-labeled murine monoclonal antibodies (mAbs) were purchased from BD, Biolegend, eBioscience, and Sungene Biotech: CD16/32(2.3G2), γδTCR (GL3), Vγ1 (2.11), Vγ4 (UC3-10A6), Vγ5 (536), CD25 (3C7), CD44 (IM7), CD62L (MEL-14), CD69 (H1.2F3), NKG2D (CX5), JAML (4E10), CCR6 (29-2L217), IL-17A (TC11-18H10.1), IFN-γ (XMG1.2). IGF-1 (H70, Santa Cruz Biotechnology, USA) was stained followed by fluorescent-labeled secondary reagents FITC goat-anti-rabbit mAb (Boster, CN), PE donkey-anti-rabbit mAb (Biolegend, USA). For surface staining, cells were first blocked with anti-CD16/32 mAb for 15 min and then incubated with different cellular surface mAbs for 30 min at 4°C. For further intracellular staining, cells were fixed and permeabilized according to the manufacturer’s recommendations (BD Biosciences, USA). For *ex vivo* detection of intracellular cytokines, epidermal cells were stimulated with 50 ng/ml PMA, 750 ng/ml ionomycin (BD Biosciences), and 100 ng/ml Brefeldin A (Beyotime, CN, USA) at 37°C, 5% CO_2_ for 6 h. For *in vitro* stimulation, eDETCs or eDETCs plus primary keratinocytes cultured with murine rIL-1β/rIL-23/rIL-17A (100 ng/ml, R&D system, USA), IL-1β/IL-23/IL-17A neutralizing Ab (10 μg/ml, BioXcell, USA), or signal inhibitors NFκB inhibitor (PDTC, 5 μM), STAT3 inhibitor (S3I-201, 100 μM) (Selleck Chemicals, USA) for 6–24 h. Cells were stained as above. Stained cells were detected on Attune Acoustic Focusing Cytometer (Life Technologies, USA) and analyzed with FlowJo software (Tree Star Incorporation, USA).

### Western Blot Analysis

Tissue protein extracts of whole skin, dermis, and epidermis were prepared by grinding the tissue into powder in liquid nitrogen. Then, the powder was lysed in lysis buffer (KeyGEN BioTECH, CN) with 1 μg/ml protease inhibitor, 5 μg/ml PSMF, and 10 μg/ml phosphatase inhibitor, followed by low-speed rotation at 4°C for 20 min and centrifugation at 14,000 × *g* for 15 min. Concentration of protein extraction was determined by BCA protein assay kit (Thermo Scientific, Rockford, USA). 25 μg/sample was run on 10% or 12% SDS-PAGE, then transferred to polyvinylidenedifluoride (PVDF) membranes (Millipore Immobilon, USA). PVDF membranes were incubated with 3% bull serum albumin (BSA) (Biosharp, CN, USA) at room temperature for 2 h, then with following Abs at 4°C overnight: IL-17A, IGF-1 (1:200, Santa Cruz Biotechnology, USA), CCL20 (1:200, R&D system, USA), IL1β/IL23 Ab (1:1,000, Abcam, UK), NF-κB p65 (D14E12), phospho-NF-κB p65 (Ser536, 93H1), STAT3 (D3Z2G)/phospho-STAT3 (Tyr705, D3A7), phospho-STAT3 (Ser727, D4X3C) (1:1,000, Cell signaling technology, USA). Anti-GAPDH Ab (1:2,000, SunGene Biotech, CN, USA) was as loading control. The membranes were visualized with goat-anti-rabbit/mouse/rat-HRP IgGs (1:5,000, SunGene Biotech, CN, USA) at room temperature for 1 h and chemiluminescence liquid for seconds (Solarbio, CN). ChemiDoc™ XRS detection system (Bio-Rad, USA) was used to analyze blots.

### HE Staining and Immunohistochemistry

Specimens of wound margin were embedded in paraffin by routine methods. The sections were stained with H&E and new epithelial length was measured under the microscope in a blinded manner. For immunohistochemistry, skin sections were deparaffinized and hydrated in xylene and graded alcohol series. Antigen retrieval was conducted by incubating sections in 10 mM citric acid (pH 6) at 95°C for 15 min. Sections were blocked and stained with goat-anti-rabbit serum or 3% BSA with 0.3% Triton X-100 (Sigma-Aldrich, Germany) at room temperature for 60 min and followed by primary antibodies: anti-IL-1β, anti-IL-23, anti-IGF-1, and anti-IL-17A (1:100–200, Abcam, UK) overnight at 4°C. Samples were washed and incubated with secondary antibodies at room temperature for 60 min. Slides were incubated with drops of diaminobenzidine solution (Boster, CN, USA) and counterstained with hematoxylin (Beyotime, CN, USA). Stained sections were examined under Olympus BX51 microscope (Tokyo, Japan).

### Immunofluorescence of DETCs and Epidermis Sheets

eDETCs were stimulated with anti-CD3ε Ab (5 μg/ml), rIL-1β, or/and IL-23 (100 ng/ml) for 30 min, then were washed with PBS. eDETCs were re-suspended by PBS to concentration of 1 × 10^6^/ml. 20 μl cell suspension was dropped to slide and went dry at room temperature. Then cells were fixation with acetone at 4°C for 30 min and washed by PBS for 5 min × 3 times. Cells were permeabilized with 0.5% Triton X-100 at 4°C for 15 min and washed three times with PBS. Cells were then incubated in 5% BSA at room temperature for 1 h. Cells were washed with PBS and incubated with anti-NF-kB p65 or anti-STAT3 Ab (1:500, Cell signaling technology, USA) in PBS at 4°C overnight. Cells were stained with DAPI (4′,6-diamidino-2-phenylindole) for 30 s and washed in PBS for 1 min. Cells were covered by mounting solution (Boster, CN, USA) and preserved at 4°C. Epidermis sheets isolated from TCRδ-GFP mice were lay on slice and directly covered by mounting solution. Slices were photographed using Zeiss Axioplan fluorescent microscope.

### RNA Extraction and Real-Time Quantitative PCR (qRT-PCR)

RNAs were extracted by a Qiagen RNeasy kit or TRIzol reagents (Invitrogen, USA). cDNA was reverse transcripted by 0.5 mg total RNA with a TaqMan reverse transcription kit (Life Technologies, USA). qRT-PCR was detected on Bio-Rad RT-PCR system using SYBR Green Supermix and gene-specific primer pairs: IL-1β: 5′—3′(forward) ACCTTCCAGGATGAGGACATGA, 5′—3′(reverse) CTAATGGGAACGTCACACACCA; IL-23p19: 35′—3′(forward) CTGAGCCACCCAGGAAAGTA, 5′—3′(reverse) TGAGAAAACCCAGAGCATCA; GAPDH: 5′—3′(forward) CGTGCCGCCTGGAGAAAC, 5′—3′(reverse) AGTGGGAGTTGCTGTTGAAGTC. Gene expression level was normalized to GAPDH and represented as fold differences by the method where *F* = 2^−Δ^**^Δ^**^Ct^, **Δ**Ct = Ct_target gene_−Ct_GAPDH_ and Δ**Δ**Ct = ΔCt_induced_−ΔCt_reference_.

### Statistical Analysis

All data were shown as mean ± SD. Statistical differences were calculated by two-tailed unpaired Student’s *t*-test or one-way ANOVE with Bonferroni comparison test on SPSS19.0 software (IBM, USA), and graphs were manufactured by GraphPad Prism software (GraphPad Software, Inc., USA). *P* < 0.05 was considered significant.

## Ethics Statement

All experiments were performed under conventional animal raising conditions in conformity with ethical guidelines and approved by the Animal Ethics Committee of the Third Military Medical University.

## Author Contributions

Conceptualization, WH; methodology, YL, YW, ML, LZ, and XH; validation, RY, JH, XZ, XH, and YH; formal analysis, YL, LZ, and YW; investigation, YL and YW; resources, GL, YJ, RW, and ZY; writing-original draft, WH, YL, and YW. Writing-reviews and editing, WH, GL, and JW; funding acquisition, WH, GL, and JW; supervision, WH, GL, and JW.

## Conflict of Interest Statement

The authors declare that the research was conducted in the absence of any commercial or financial relationships that could be construed as a potential conflict of interest.

## Supplementary Material

Figure S1 The number of Vγ4 T cells was decreased in dermis rather than draining lymph nodes. Wild-type and Vγ4 T-cell depletion mice were sacrificed on days 0, 3, 5, and 7 after excision. **(A)** Dermal sheets around wounds were collected from 3 mice with 4 wounds/mice. Percentage of dermal Vγ4 T cells was analyzed by FACS. **(B)** Percentage of Vγ4 T cells in draining lymph nodes was analyzed by FACS. Representative data of three individual experiments were shown. Dots represent individual animals. All error bars represent mean ± SD. *P* value was assessed by one-way ANOVA with Bonferroni’s comparison test (***P* < 0.01, ****P* < 0.001).

Figure S2 Wound healing was defected in IL-17A knock out mice. Age- and sex-matched C57BL/6 wild-type mice and *Il-17a*^−/−^ mice were anesthetized and full-thickness wounds were then generated using a sterile 6 mm punch tool. Wound-closure kinetics was measured over time and dots in wound closure kinetics represent 10 wounds per group (2 wounds/mouse, 5 mice/group). Bars represent mean ± SD. All data were representative of three independent experiments. *P* value was calculated by Student’s unpaired *t*-test (***P* < 0.01, ****P* < 0.001).

Figure S3 IL-17A production on dendritic epidermal T cells (DETCs) was significantly increased by IL-1β and IL-23. eDETCs were stimulated for 6 h with anti-CD3ε, anti-CD3ε plus rIL-1β, anti-CD3ε plus rIL-23, anti-CD3ε plus rIL-1β and rIL-23, anti-CD3ε plus rIL-1β and rIL-23 with NF-κB inhibitor (PDTC), or anti-CD3ε plus rIL-1β and rIL-23 with STAT3 inhibitor (S3I-201) in the presence of brefeldin A. IL-17A expression on eDETCs was detected using FACS. Data are representative of three individual experiments. All error bars represent mean ± SD.

Figure S4 IL-1β and IL-23 accelerated IL-17A production in epidermis. Cell suspensions were isolated from epidermis of normal skin **(A)** or wound margin **(B)** on day 3 post excision (4 wounds/mice, 3–5 mice/group). Cells were stimulated with anti-CD3ε Ab, rIL-1β, and IL-23, or anti-CD3ε Ab + rIL-1β + IL-23 for 6 h *in vitro*. Rectangles in the upper lane represent for Vγ5T cells and rectangles in the down lane represent for IL-17A positive population of Vγ5T cells gated in the upper rectangles. Statistical figures are on the right side. Data are representative of three individual experiments. All error bars represent mean ± SD. *P* value was assessed by one-way ANOVA with Bonferroni’s comparison test (**P* < 0.05, ***P* < 0.01, ****P* < 0.001).
