# Supplementary material for: Vγ4 T Cells Inhibit the Pro-healing Functions of Dendritic Epidermal T Cells to Delay Skin Wound Closure Through IL-17A
Source: Front Immunol. 2018 Feb 12;9:240. doi: 10.3389/fimmu.2018.00240 (PMC5816340; doi:10.3389/fimmu.2018.00240)
Supplement: Figure S1 — The number of Vγ4 T cells was decreased in dermis rather than draining lymph nodes. Wild-type and Vγ4 T-cell depletion mice were sacrificed on days 0, 3, 5, and 7 after excision. (A) Dermal sheets around wounds were collected from 3 mice with 4 wounds/mice. Percentage of dermal Vγ4 T cells was analyzed by FACS. (B) Percentage of Vγ4 T cells in draining lymph nodes was analyzed by FACS. Representative data of three individual experiments were shown. Dots represent individual animals. All error bars represent mean ± SD. P value was assessed by one-way ANOVA with Bonferroni’s comparison test (**P < 0.01, ***P < 0.001). [file Image_1.PDF]

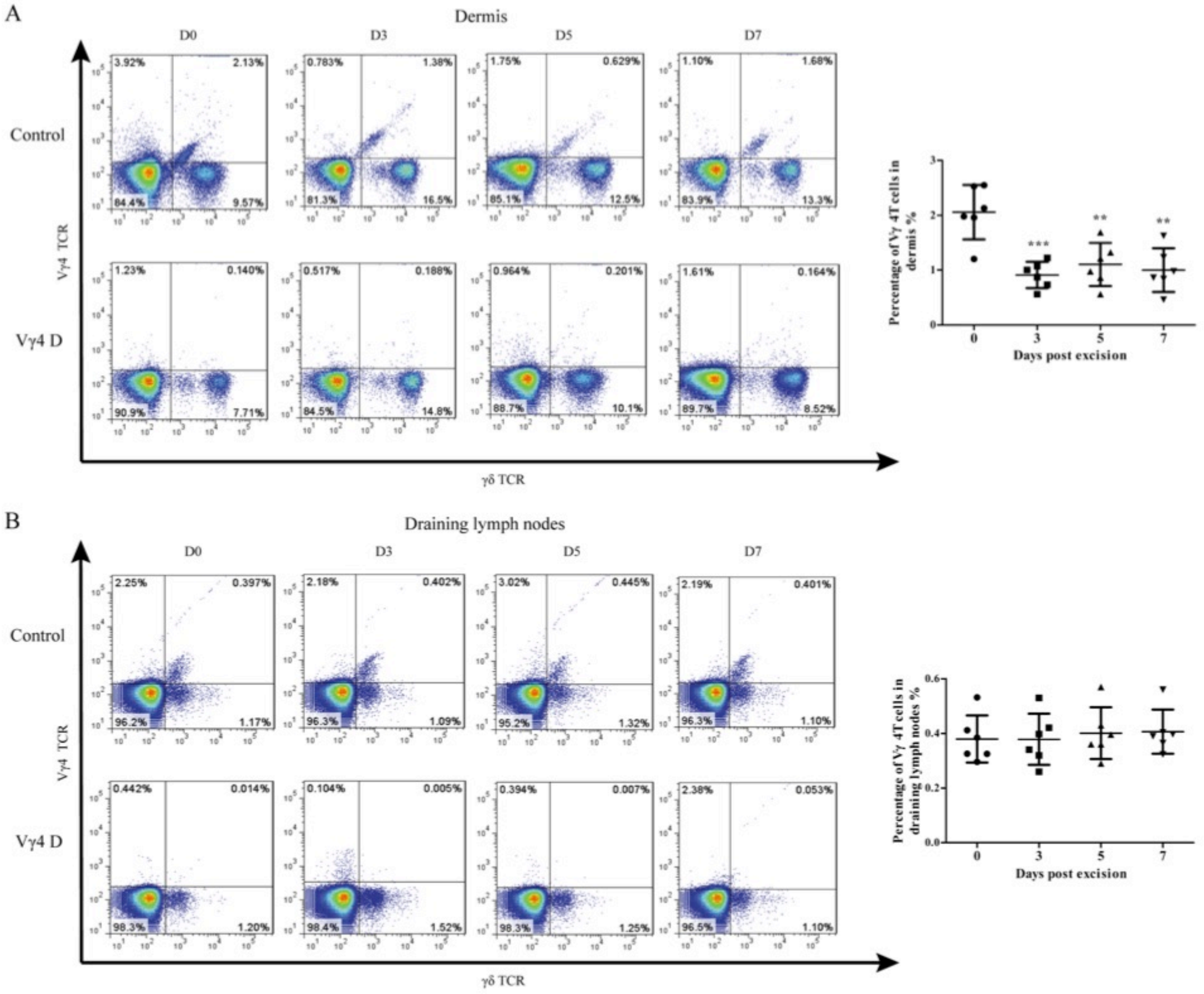

Supplementary Figure S1

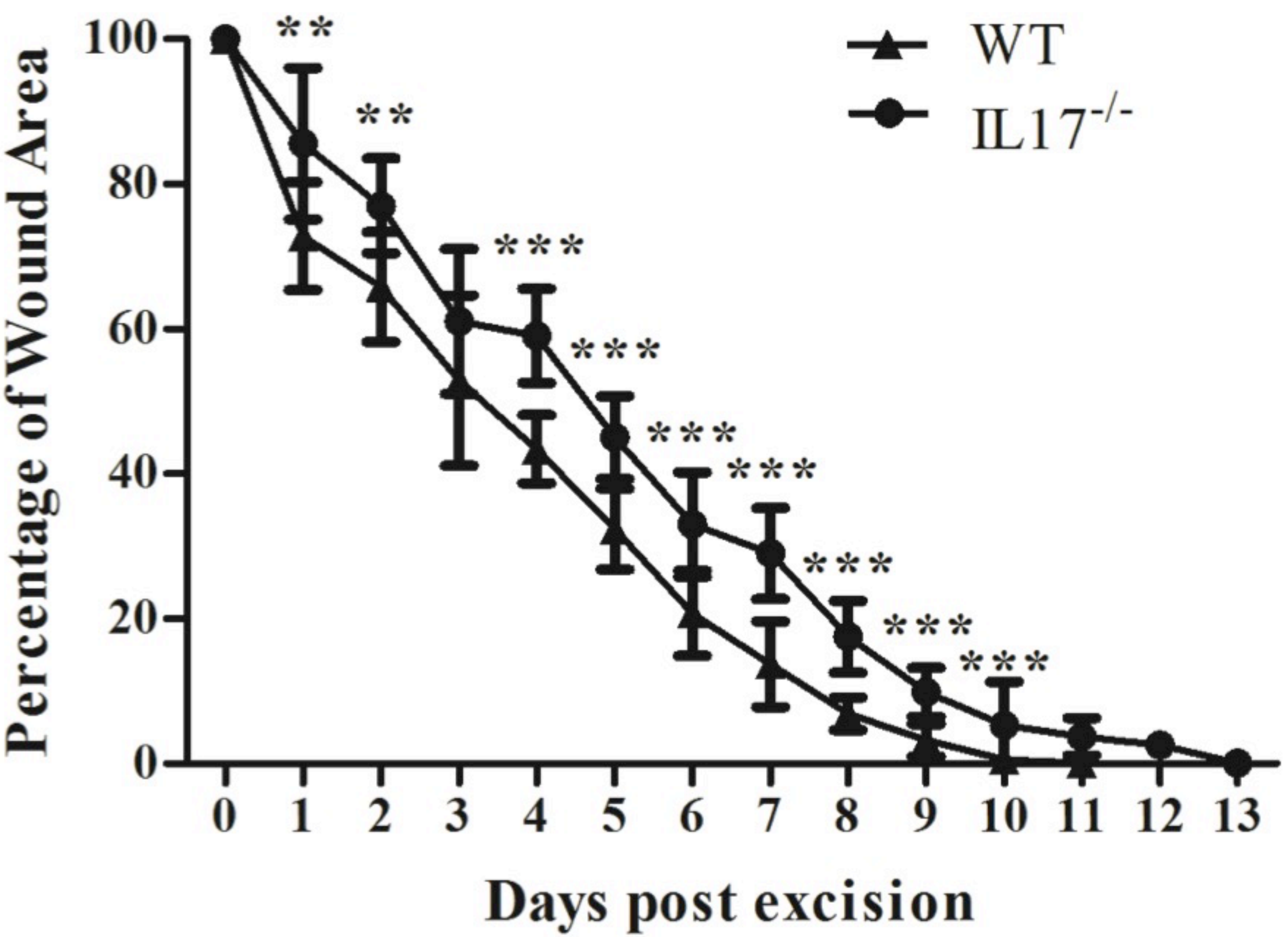

Supplementary Figure S2

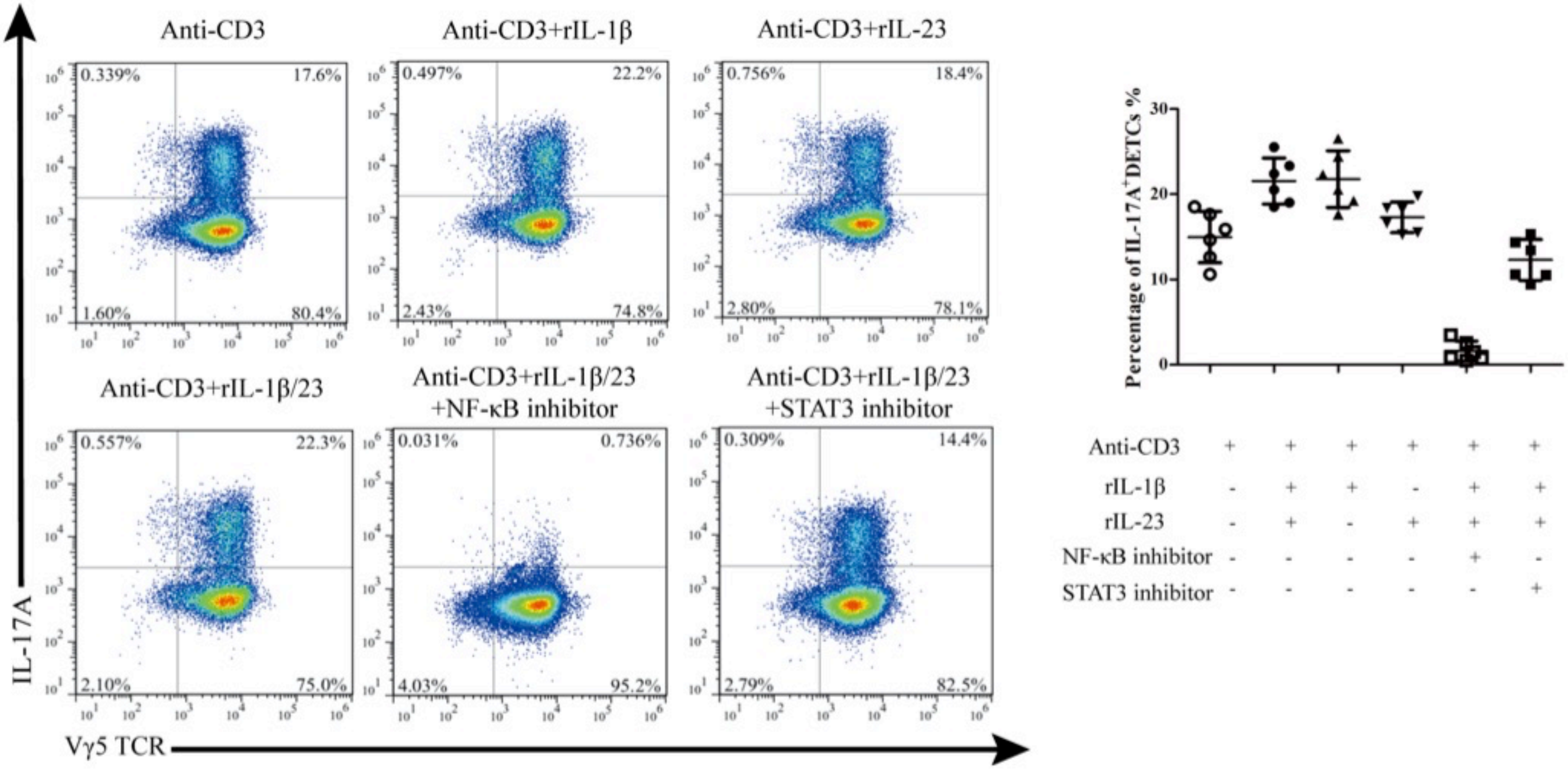

Supplementary Figure S3

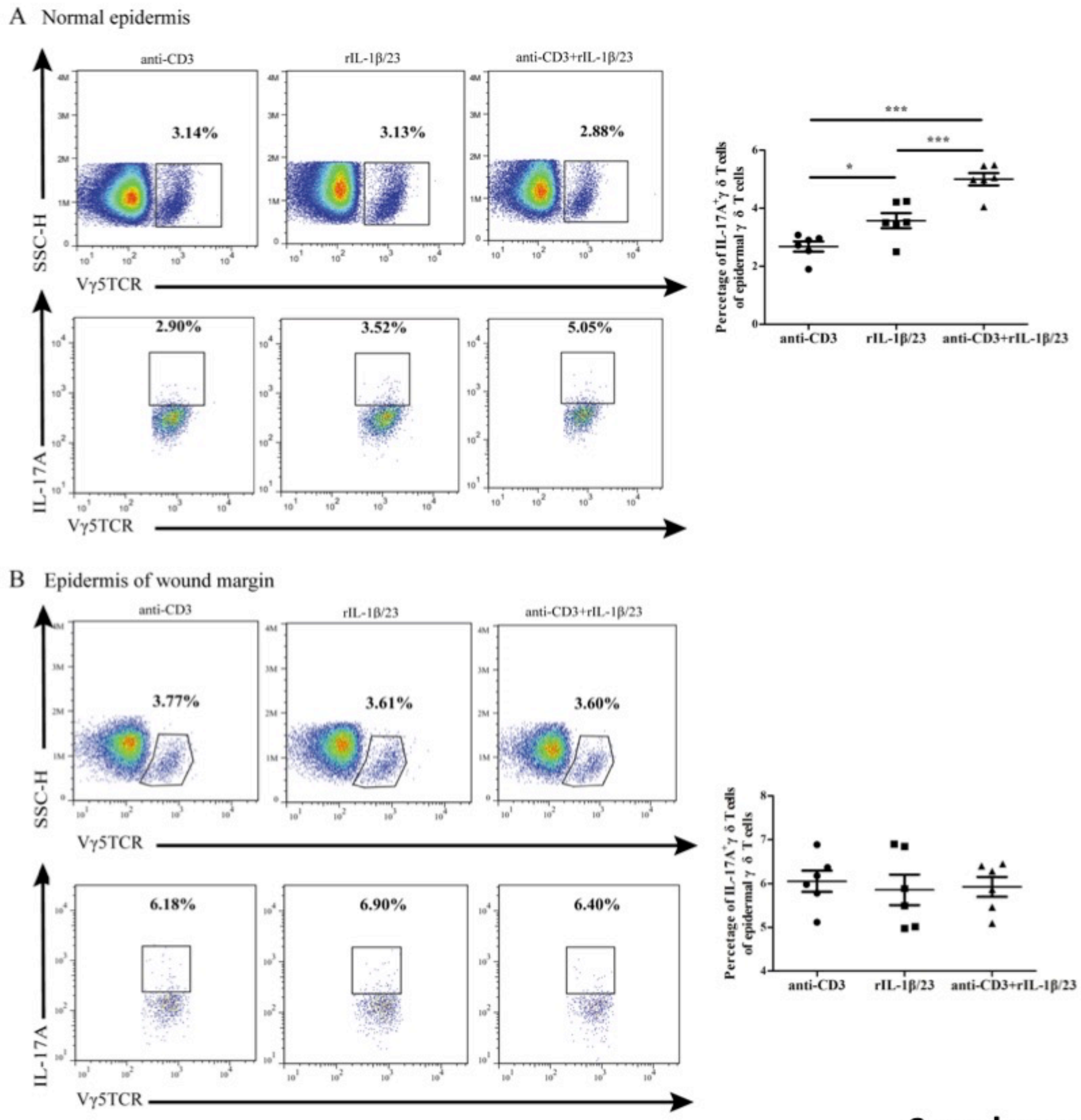

Supplementary Figure S4
